# Responding to Families Who Express Biases: An Adaptable Standardized Participant Communication Simulation to Train Upstander Pediatric Providers

**DOI:** 10.15766/mep_2374-8265.11588

**Published:** 2026-03-27

**Authors:** Kelly L. Corbett, Michelle Tyler, Frances Lim-Liberty, Juhi Rattan, Carol-Lynn O'Dea

**Affiliations:** 1 Assistant Professor, Department of Pediatrics, Geisel School of Medicine at Dartmouth; Section of Critical Care Medicine, Dartmouth Health Children's; 2 Assistant Professor, Department of Pediatrics, Geisel School of Medicine at Dartmouth; Program Director, Neonatology-Perinatal Medicine Fellowship, Dartmouth Health Children's; 3 Assistant Professor, Department of Pediatrics, Director of Pediatric Clerkship, Geisel School of Medicine at Dartmouth; Section of Endocrinology, Dartmouth Health Children's; 4 Third-Year Pediatric Critical Care Fellow, Hassenfeld Children's Hospital at NYU Langone Health, NYU Grossman College of Medicine; 5 Assistant Professor, Department of Pediatrics, Geisel School of Medicine at Dartmouth; Program Director, Pediatric Residency, Dartmouth Health Children's

**Keywords:** Bias, Racism, Discrimination, Communication Skills, Diversity, Equity, Inclusion, Faculty Development, Professionalism, Simulation, Standardized Patient

## Abstract

**Introduction:**

Pediatricians must navigate family dynamics, including addressing biases, while modeling appropriate behavior in front of a pediatric patient. We developed an adaptable anti-bias simulation workshop involving standardized participants (SP) as the biased parent of a pediatric manikin patient.

**Methods:**

The workshop was originally designed for pediatric residents, and was adapted for faculty and neonatology fellows. The 60-minute simulation workshop included 3 short, escalating cases of discriminatory behavior toward a member of the medical team. Biased behavior included overt racism and transphobia. The participants were required to develop a therapeutic alliance with the parent, de-escalate the situation, and model appropriate anti-biased behavior in front of an observant pediatric patient. After each simulation, learners debriefed with the facilitator, peers, and the SP. Program evaluation was conducted by anonymous pre- and postworkshop surveys.

**Results:**

Thirty-four participants completed the workshops: 16 residents (80% of the residency), 13 faculty members, and 5 neonatology fellows. All participants met educational objectives during the simulation. In the preworkshop survey, 26% of participants agreed that they had the tools to respond to discriminatory behavior; after the workshop, 100% of participants agreed (*P* < .001). Confidence to appropriately respond to discrimination improved after the simulation. The workshop is now integrated into residency annual education.

**Discussion:**

We implemented our upstander simulation workshop to train learners to address patients’ families who direct discriminatory behavior toward health care team members. Strengths of the program included working with trained SPs and the inclusion of the pediatric manikin patient to reflect realistic clinical encounters.

## Educational Objectives

By the end of this session, learners will be able to:

1. Apply effective communication strategies, establish a therapeutic alliance, and de-escalate patients’ family members who exhibit bias toward members of the health care team.
2. Model anti-bias language in front of pediatric patients as upstanders.
3. Advocate for an inclusive, supportive clinical environment for the entire health care team, without excluding targeted individuals.

## Introduction

Discrimination and racism are “painful and degrading indignities, which cumulatively contribute to moral distress and burnout.”^1^ In a national survey of 529 physicians, survey responses indicated that physicians who experienced racial or ethnic discrimination at work had twice the odds of leaving the workplace.^2^ In recent years, there have been increasing reports of medical trainees receiving mistreatment from patients or family members, including requesting a different provider based on race or ethnicity.^3–7^ Medical trainees are a vulnerable population, who balance hierarchical expectations and patient-centered care, and must still maintain personal self-care. In a multicenter survey study, only one-third of residents (73 of 231 respondents) indicated that they were confident in how to respond to bias from patients or families, while 89% of residents (206 of 232 respondents) identified anti-bias training as necessary for residents and faculty.^7^

Residents need to learn how to respond to biases, not only for their own well-being, but also as leaders who must support their multidisciplinary teams. Residents are trainees, but they are also supervisors of students and junior house-staff. Supporting the entire team is an important responsibility for the team leader. Anti-bias training can dispel the idea that tolerating discriminatory behavior is expected and acceptable.^8^ Residents need to learn how to transform from passive bystander to active upstander when encountering families expressing biased behavior toward the medical team.

There have been increasing calls for curricula to teach trainees how to appropriately respond to discriminatory behavior during clinical encounters.^5,7–10^ There are several recent *MedEdPORTAL* publications, such as the RISE UP virtual and role-playing curriculum,^9^ the VITALS microaggression curriculum,^10^ and the ERASE faculty workshop to coach faculty to assist trainees experiencing bias.^11^ Eisenberg and Kieffer published a simulation for internal medicine residents who experience bias directly.^5^ Our simulation aimed to build on these prior workshops by training pediatric residents to become active upstanders when faced with overt racism or transphobia from families. Our workshop included communication practice with trained standardized participants (SPs) within the immersive simulation learning environment for added realism.

At our institution, medical student and resident mistreatment from families prompted us to review published upstander training programs to identify strategies to manage these difficult behaviors. We developed an adaptable anti-bias simulation workshop involving an SP as the biased parent of a pediatric manikin patient. Our educational objectives were adapted from the communication frameworks previously described by Whitgob et al^3^ and Shankar et al,^12^ which emphasized building a therapeutic alliance with the family and ensuring trainee safety. Our workshop added the educational objective of modeling anti-bias behavior for observant pediatric patients. The workshop was grounded in transformational learning theory, by creating a disorienting experience in the first case, followed by critical reflection with structured debriefing, and skill acquisition through deliberate practice with repeated exposure, achieved through 3 short simulated cases of escalating discriminatory behavior toward a member of the medical team.^13,14^

## Methods

### Development

We decided to work with trained SPs, rather than participant role-playing, to provide learners with immersive realism, gain feedback from the patient's family perspective, and facilitate a brave space for learners to practice without fear or embarrassment from role-playing with their peers.^13,15,16^ To maintain trainee psychological safety, we wrote the cases for the parent to discriminate against a member of the medical team, but not the participants directly. Our workshop was designed for participants to practice using anti-bias de-escalation language as upstanders. We provided participants with a scripted language tool for assistance, which offered suggested verbal phrases compiled from those described by Whitgob et al^3^ and Shankar et al^12^ (Appendix A). The simulation scripts were developed by the lead author (Kelly L. Corbett), a pediatric intensivist who oversees the resident simulation curriculum. The early versions were piloted for feasibility and flow with a co-author (Juhi Rattan), a pediatric resident. Feedback from the simulation educators and SPs during piloting was incorporated to strengthen the scripts for each case (Appendices B–F).

This curriculum was reviewed by the Dartmouth Health Office of Diversity, Equity, Inclusion and Belonging. Based on feedback from this review and the importance of debriefing challenging encounters, we added the simulated debrief following the interaction between the participant and the second SP (SP2) in the third case.

### Participants

The anti-bias simulation workshop was originally designed for pediatric residents and was incorporated into the academic half-day curriculum. The workshop was delivered to each small group of 5–6 residents, and repeated weekly over a 4-week academic block to include all the residents. Each small group consisted of a mix of PGY 1–3 residents. We began the anti-bias simulation curriculum in academic year 2022–2023, and we have now run it for 4 consecutive academic years.

The workshop was developed to be adaptable. With minimal adjustment to the case scripts, the workshop was delivered in 2024 to the neonatology fellows, and delivered to the pediatric faculty during the annual educators’ retreat (Appendix G).

### Setting

The Patient Safety Training Center (PSTC) is a moderate-sized academic simulation center with space and resources for multidisciplinary, experiential learning. The PSTC coordinates and trains SPs who have experience in communication training with health care professionals. Live-stream video was transmitted from the simulation room into the debriefing room to allow observation of the case and include nonparticipants in each debrief. If the live-stream option is unavailable at other facilities, the nonparticipants can usually observe in the simulation control room, if space allows.

### Equipment

We used a Laerdal SimMan 3G manikin as the teenage patient in the case, which has functionally blinking eyes and speakers to provide interactive speech. Because the manikin is blinking and observant, the participants are directed during the prebrief that the manikin can hear and speak, and should be included in the discussion. If a high-fidelity manikin is unavailable, a low-fidelity manikin can be used instead, and the prebrief should emphasize the need to suspend disbelief and still include the child in the dialogue. Although not required, it is helpful for the first SP (SP1) to wear an ear-piece so the facilitator can provide real-time instruction as needed. When the cases were adapted for the neonatology fellows, the teenager Laerdal SimMan 3G manikin was replaced with a SimBaby manikin.

### Personnel

The workshops were facilitated by a minimum of 1 experienced simulation educator from within the residency leadership (Kelly L. Corbett, Michelle Tyler, Frances Lim-Liberty, Carol-Lynn O'Dea) using a facilitator guide (Appendix G). In general, the workshop can be facilitated by leaders without additional anti-bias professional training. During the simulation, the manikin's voiced replies were provided in real-time by the facilitators. The SPs (2 per workshop) were recruited by the PSTC and received a 1-hour (virtual or in-person) preparation session (Appendix H) prior to the workshop. The manikin patient was medically stable and did not have multiple states to transition through. As such, a simulation technician prepared the equipment prior to each workshop, but was not required to be present in the control room during the workshop.

### Implementation

A 30-minute didactic session (Appendix I) was conducted a week before the simulation workshop during a noon conference to introduce the anti-bias communication framework and the scripted language tool for de-escalation. For the 1-day events for the faculty and the neonatology fellows, this didactic was given just prior to the simulation prebrief.

On the day of the workshop, we gave a 5-minute prebrief to review the brave space concept in the simulation center.^16^ Brave spaces move beyond an overreliance on safety to create a space where difficult or uncomfortable conversations can be held, with the goal to advance social justice.^16^ Facilitators warned that the cases used aggressive language, including shouting.

The workshop included a total of 3 short, escalating cases, lasting a total of 60 minutes. Cases 1 and 2 (Appendices B and C) were each about 5 minutes in the room, and Case 3 (Appendices D, E, and F) was 10 minutes, allowing deliberate practice with escalating cases.

Case 1 (Appendix B) involved the SP parent (SP1) of the pediatric manikin patient using racist language toward a medical student who was a person of color but not present in the room, and was designed for learners to practice how to respond to racism directed at other team members. The language used by the SP was racist, above the level of microaggressions.

Case 2 (Appendix C) was similar to the first case, but the SP parent was much more overtly aggressive and racist, and was designed for the learners to apply the feedback received after Case 1.

Case 3 (Appendices D–F) involved an SP2 acting as a gender nonbinary team member who was directly targeted with transphobic language from the parent (SP1), and was designed for learners to build on the de-escalation skills practiced in Cases 1 and 2. The participants needed to de-escalate SP1 and support SP2. Upon leaving the simulation room, the participants were directed to practice debriefing the event with SP2 before concluding the case. One of the teaching points for Case 3 was for the participants not to exclude targeted team members, which risks undermining the individuals’ agency and contributes to implicit agreement with the bias.^3,4^

The SP acting as a parent was directed to escalate if the participants failed to develop a therapeutic alliance, and to de-escalate if the participants appropriately used anti-biased language. A total of 1–2 learners participated in each case, while the remaining small group observed the case and joined for the debrief.

### Debriefing

After each simulation, learners debriefed with a trained facilitator, peers, and the SP, following the Promoting Excellence and Reflective Learning in Simulation (PEARLS) structured framework.^17^ Between Cases 1 and 2 there were abbreviated 10-minute feedback debrief sessions (Appendix G), with the main 20-minute debrief for the workshop occurring at the end of Case 3 (Appendix G). During the debriefs, the SPs were invited to share their impressions as the parent, and to express how the participants’ language had been received. After the final case, SP2 was encouraged to give feedback on how effective the participants’ language was on supporting the medical team member, and particularly if the participants used the wrong pronouns.

### Assessment

Program evaluation was conducted by anonymous pre- and postworkshop surveys using a 5-point Likert-style questionnaire (1 = *strongly agree*, 5 = *strongly disagree*; Appendix J). The surveys were written by one of the authors (Kelly L. Corbett) and reviewed internally by residency leadership (Michelle Tyler, Frances Lim-Liberty, Carol-Lynn O'Dea), with the primary goal being program evaluation, rather than a goal to validate a new data collection tool. Pre- and postworkshop survey responses were not linked. We compiled the survey responses and analyzed the data using the Fisher exact test to compare changes before and after the training, given the small sample size and ordinal data. All analyses were performed using R, version 4.3.2. We did not collect or report data on gender, race, or ethnicity, to maintain participant anonymity. We collected informal written qualitative feedback after each session by open-response questions on the surveys. The study was reviewed by the Dartmouth Health Institutional Review Board and was determined not to be human subject research.

## Results

A total of 34 participants completed the simulation workshop and the postsimulation evaluation. Sixteen pediatric residents completed the training in March 2023, and all completed the program evaluation, representing 80% of the residency program. In June 2024, the workshop was delivered to the pediatric faculty during the annual educator retreat. Fourteen attending faculty participated in the simulation workshop; 1 of the 14 did not complete the postworkshop survey. In November 2024, the workshop was adapted for the neonatology fellows, and 5 participated and completed the program evaluation.

On the preworkshop survey, there was a range of views on how prevalent discrimination was in the clinical environment. In total, 63% (10/16) of the residents, 40% (2/5) of the neonatology fellows, and 93% (13/14) of the pediatric faculty *agreed* or *strongly agreed* with the statement that “discriminatory behavior is prevalent in the clinical learning environment.” Only 19% (3/16) of residents had previously received training on managing discriminatory behavior from families, whereas 43% (3/5) of neonatology fellows and 43% (6/14) of faculty pediatricians had prior training.

During the cases, all participants were successful at meeting the educational objective of establishing a therapeutic alliance and de-escalating the biased parent. None of the participants excluded SP2 from the third case.

On the preworkshop survey, 26% of participants (31% of residents, 29% of faculty, and 60% of fellows) *agreed* or *strongly agreed* that they had the tools and language to respond to discriminatory behavior in the clinical setting; after the workshop, 100% of participants *agreed* or *strongly agreed* that they had the tools to respond to discriminatory behavior (*P* < .001). When this perceived capability was analyzed by level of training, a statistically significant improvement in scores was observed in the residents and faculty after the training session (*P* < .001), but no significant change in scores was observed for the fellows (*P* = .44).

The number of participants in aggregate who *agreed* or *strongly agreed* that they were confident in addressing bias from patients’ families improved from 23% pretraining to 94% posttraining (*P* < .001). This improvement in confidence was statistically significant for the residents and faculty members (*P* < .001), but the change did not reach significance for the fellows (*P* = .05) due to the small sample size (Figure).

**Figure.**
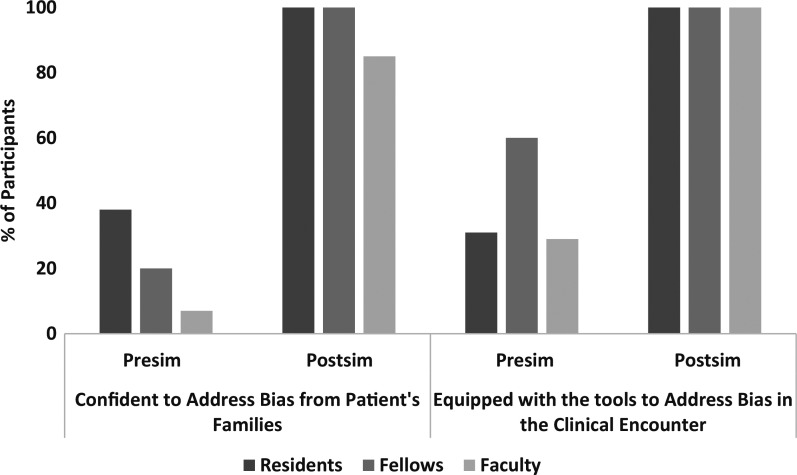
Percentage of pediatric residents (*n* = 16), neonatology fellows (*n* = 5), and pediatric faculty members (*n* = 13) who agreed or strongly agreed with statements regarding their confidence to address bias in families and the clinical encounter, as indicated on evaluation surveys before and after participation in the anti-bias simulation (Sim) workshop. Resident data from March 2023. Pediatric faculty data from June 2024. Neonatology fellow data from November 2024.

On the postworkshop survey, 94% of the residents, 100% of neonatology fellows, and 100% of faculty *agreed* or *strongly agreed* to having learned a new skill during the training. All participants (residents, faculty, and neonatology fellows) agreed that the training was high quality and relevant to their professional development.

Postsimulation comments were positive and residents requested repeating the training annually. Comments from all groups appreciated the SP involvement, with 1 participant describing it as “impressive acting, felt real, very unique experience.” Participants reflected on specific aspects that were helpful, such as practicing appropriate pronoun use as exemplified in the comment: “the last scenario was very valuable for me as I want to be respectful but do struggle with remembering pronouns.”

## Discussion

We developed this simulation workshop involving SPs to train learners to address patients’ family members who direct discriminatory behavior toward members of the health care team. We implemented our simulation workshop to practice anti-bias language and to exercise modeling anti-bias responses in front of pediatric patients. Participants reported having the tools and language as well as feeling more confident in addressing discriminatory behavior in front of an observant pediatric patient. Strengths of the program included working with trained SPs, and the inclusion of the pediatric manikin patient to reflect realistic pediatric clinical encounters. The workshop has now been integrated into residency annual education for the past 4 academic years.

There are currently several published anti-bias educational curricula.^5,9–11^ Our workshop adds to the growing body of educational work by including the realism of SPs in an adaptable format that can be adjusted for participants across different pediatric settings. The SPs were viewed as a valuable asset by the participants for the increased realism during the cases and the focused feedback during the debriefs. This format worked well with small groups for personalized interaction with SPs, but would be harder to incorporate into larger group training events.

We did not want any of the participants to feel directly targeted during the simulation, and adapted the biased language toward identity groups that did not represent the participant. This requires knowledge of the self-reported identities of the participants in advance to ensure their psychological safety.^13^ Our center is small and this was feasible, but adapting this to a larger group may prove difficult and runs the risk of triggering participants who encounter biased language during the cases. Facilitators must be prepared to support participants from unintended consequences.

One of the benefits of SPs in simulation is to reduce the risk to real patients (or participants) during practice, but such simulations can shift the risk of potential harm onto SPs instead.^18^ Efforts to teach cultural humility and diversity risk negative effects on SPs such as tokenism, stereotyping, and re-traumatization, causing psychological harm to SPs.^19^ Our goal to ensure a safe space for our participants was balanced with our efforts to mitigate harm to SPs. The first 2 cases were written to refer to a BIPOC student who was not present in the encounter, rather than subject an SP to the derogatory language in the case. Comments from the participants requested including an SP in this role, but recruiting an SP to be a target of biased behavior ran the risk of tokenism and perpetuating racial injury.^18^ To allow for the escalation of biased behavior against a present SP in the third case, we developed the case for gender identity bias, crafting language with input from a representative of the LGBTQ community.^19^ Affirmed pronouns are not visible and recruiting SPs for the SP2 role included gender-diverse participants or cisgender individuals who felt comfortable with the role. After the case debriefs for the participants, we also debriefed and de-roled (the act of detaching from the role) the SPs given the stressful environment of the cases.^13,18^ The SP1 role of the biased parent was also difficult, and several SPs made an effort during the debriefs to disavow the racist and hateful language they had delivered during the simulation.

Pediatricians are uniquely positioned to embrace an antiracist framework with families and model appropriate behavior in front of pediatric patients.^20^ Children are not born racist, but develop an understanding of racial construct by experiences, and pediatricians standing up to racism can challenge previous racist narratives to which the child has been exposed.^6^ By speaking up in the face of racism or bias, pediatricians avoid implicit agreement. The kids are listening.

There are several limitations with our approach. As a single-center implementation with a small sample size, external generalizability is limited. While all participants demonstrated competence and achieved the educational objectives during the simulations, our evaluation metrics focused on postworkshop self-reported assessments. Participants reported gaining new skills, confidence, and tools for future encounters with biased families, but we did not evaluate retention or application of skills in situ. This limits the evaluation to Kirkpatrick Levels 1 and 2.^21^

Each iteration of the workshop helped refine it, particularly in the richness of the debrief dialogue from resident reflections. Many residents shared their personal experiences with biased behavior and brought to the discussion insight into how to grow as an upstander. These discussion points have been added to the Facilitator Guide (Appendix G, under “Common Discussion Topics”). Residents requested that in the future we develop this curriculum toward addressing microaggressions in cases with SPs. Other future directions include expanding to include multidisciplinary team members who interact with families, reinforcing an anti-bias culture across the spectrum of health care.

## Appendices


Scripted Language Tool.docxCase 1 - Inpatient.docxCase 2 - Inpatient.docxCase 3 - Inpatient_SP1.docxCase 3 - Inpatient_SP2.docxCase 3 - Simulation.docxFacilitator Guide.docxSP Educator Training Notes.docxAnti-bias Intro Presentation.pptxPre- and Postsurveys.docx
*All appendices are peer reviewed as integral parts of the Original Publication.*
