# Supplementary material for: Responding to Families Who Express Biases: An Adaptable Standardized Participant Communication Simulation to Train Upstander Pediatric Providers
Source: MedEdPORTAL. 2026 Mar 27;22:11588. doi: 10.15766/mep_2374-8265.11588 (PMC13021565; doi:10.15766/mep_2374-8265.11588)
Supplement: Supplementary file 1 — Scripted Language Tool.docxCase 1 - Inpatient.docxCase 2 - Inpatient.docxCase 3 - Inpatient_SP1.docxCase 3 - Inpatient_SP2.docxCase 3 - Simulation.docxFacilitator Guide.docxSP Educator Training Notes.docxAnti-bias Intro Presentation.pptxPre- and Postsurveys.docx [file mep_2374-8265.11588-s001.zip › A. Scripted Language Tool.docx]

**Scripted Language for addressing a discrimination from a patient or family:**

1. **Ensure the patient is clinically stable. Redirect the goal to the medical visit**

- “Let’s focus on your child’s health”
- “I first need to make sure your child is OK before we can discuss XYZ”
- “All members of your medical team share the same goal with you, to provide the best care possible so your child can get better”
- “I’m worried about your child’s symptoms/disease, and need to ask you to keep your comments respectful so we can stay focused on the goal to help your child feel better”

1. **Inquire further into the biased statements. Use “I” statements as able**

- “I can see you’re upset. Tell me what you are afraid of.”
- “Are you aware that your comments are making the staff/team uncomfortable?”
- “When you say ‘xyz’, can you tell me more about what you mean?”
- “I believe you were trying to compliment Dr. ABC, but what you said made me/us feel uncomfortable”
- “I want you to know that I/we felt disrespected by that comment”
- “I’m not sure if you were aware, but that could be interpreted as a racist comment”
- “I’m not sure I understand your comment. It feels like you are discriminating against Dr. ABC because of their race/religion/gender/accent.”

1. **Address the comment. State that it is inappropriate**
   - “I understand that this can be a stressful situation, but I need to ask you to keep your comments respectful”
   - “I’m surprised that you thought that would be an appropriate comment/joke.”
   - “We expect patients and staff to be treated with respect in this unit/hospital. We cannot tolerate that kind of language”
2. **Support the team members in front of the patient/family:**
   - “I respect every member of your medical team, and ask you to do the same”
   - “You’re very lucky to have Dr. ABC on your team—they are an exceptionally talented and caring provider”
   - “This is a teaching hospital with students and resident-physicians from a diverse range of backgrounds – all of whom are fully qualified to take care of you and your child.”
   - “I would trust Dr. ABC with my own child’s care.”
   - “Dr. ABC is the physician in charge of your child’s day-to-day care.”
3. **Ensure team safety**
   - If the behavior continues or escalates, it may be necessary to remove from the situation for a cooling off period: “We are going to come back in 30 minutes and hope that you will be ready to focus on your child’s health at that time”
