# Supplementary material for: Responding to Families Who Express Biases: An Adaptable Standardized Participant Communication Simulation to Train Upstander Pediatric Providers
Source: MedEdPORTAL. 2026 Mar 27;22:11588. doi: 10.15766/mep_2374-8265.11588 (PMC13021565; doi:10.15766/mep_2374-8265.11588)
Supplement: Supplementary file 1 — Scripted Language Tool.docxCase 1 - Inpatient.docxCase 2 - Inpatient.docxCase 3 - Inpatient_SP1.docxCase 3 - Inpatient_SP2.docxCase 3 - Simulation.docxFacilitator Guide.docxSP Educator Training Notes.docxAnti-bias Intro Presentation.pptxPre- and Postsurveys.docx [file mep_2374-8265.11588-s001.zip › B. Case 1 - Inpatient.docx]

Appendix B: *Case 1 - Inpatient*

Date: 12/21/22

Primary Case Author: Kelly Corbett, MD

Secondary Case Author: Juhi Rattan, MD

Standardized Patient Educator: Peter Thurber

Name of Case: Antibias hybrid sim, SP case 1

Name of Educational and/or Assessment Activity: resident antibias training workshop

Patient Name: “Billy” is the teenager manikin. The SP is the parent “John/Jane” (or other guardian as necessary, dependent on the age of the SP)

Chief Complaint: short of breath related to vaping

Challenge Question: Maintain professionalism and improve communication skills when faced with difficult interactions with biased family members targeting members of the healthcare team. The SP will start “haggling” to have the medical student removed from the medical team. The SP will become emotional and angry unless the participants appropriately handle the situation, and will escalate the language and behavior if the learners do not directly address the biased behavior

**Domains: Check all that apply**

- Professionalism

X **Communication and Interpersonal Skills**

- Medical History
- Physical Exam
- Shared Decision-Making
- Patient Education
- Clinical Reasoning
- Documentation
- Handoff
- Presentation
- Other:

**Type and Level of Learner**: Pediatric residents, pgy1-3

**Case Objectives**: Please list specific objectives for each of the domains you have checked above:

1. Apply effective communication strategies, establish a therapeutic alliance, and de-escalate patients’ family members who exhibit bias toward members of the healthcare team

2. Model antibias language in front of pediatric patients as upstanders

3. Advocate for an inclusive, supportive clinical environment for the entire healthcare team, without excluding targeted individuals

| SETTING: outpatient, in patient, ED, home, nursing home, rehab, group, etc. | PICU (inpatient) |
| --- | --- |
| PATIENT PROFILE: Information about the “patient” that helps select an SP and helps the learner get an understanding of them as a person. SP will know more information about the patient than learner will ever ask but allows SP to portray a fully developed patient personality. If none of the items below are particulars for the case, please write “all may be used.” | |
| Age range | Adult. If SP is older than parent-age, adjust the script to be the grandparent guardian of the manikin patient |
| Religious/spiritual background | all |
| Sex (e.g., male, female, intersex, transwoman, transman) | Male or female |
| Sexual orientation (e.g., heterosexual, lesbian, gay, bisexual, pansexual, queer, asexual) | Hetero |
| Gender expression (e.g., man, woman, genderqueer) | Cis (male or female) |
| Race and ethnicity (e.g., to promote educational diversity, we use a diverse pool of SPs.) | White (case is written for the SP to be biased against a BIPOC medical team member; if the SP parent is a person of color, adjust the bias in the case (against gender, ethnicity, religion) |
| Physical description (e.g., BMI, height range) | All |
| Physical limitations | none |
| Patient appearance (e.g., disheveled, hospital gown, business casual, casual) | Casual |
| Moulage + location (e.g., none, bruises, scars, body piercing, tattoos) | NA |
| Affect (e.g., pleasant, cooperative) | Disgruntled, frustrated, against the medical student (not present) who is a person of color |
| Family group (e.g., who is family, who they live with) | Parent to the manikin-patient |
| Education | All |
| Level of health literacy | All |
| Employment, if any - present and past, noting any current stresses | NA |
| Home/homeless - type of dwelling, number of stories, owned or rented | NA |
| Financial situation - any current stresses | NA |
| Insurance status (e.g., un/under/insured, public/private, HMO/PPO) | NA |
| Habits (i.e., diet, exercise, caffeine, smoking, alcohol, drugs) | NA |
| Activities (i.e., hobbies, sports, clubs, friends) | NA |
| Typical day - what is the usual daily routine | NA |

| CASE INFORMATION | |
| --- | --- |
| Chief Concern: What the patient will say when greeted by the student. The patient’s primary reason for seeking medical care often stated in their own words. | Initially relieved that the participants are “not that student again” and then express frustration/anger to ask the participants to make sure “that student” is not allowed to come in the room any more |
| Additional Concerns: Other, if any, concerns the patient has today (i.e., symptoms, requests, expectations, etc.) that will become part of set agenda. | If the residents try to build understanding with the SP, they learn that the parent is very stressed and scared about their child’s respiratory failure |
| THE PATIENT’S STORY: The SP will be asked to tell their symptom story and the personal and emotion impact for each of their concerns. You will want to write this in the patient’s voice. The symptom story should be able to answer this question: “Tell me more about [chief concern/additional concern], starting at the beginning and bringing me up to now.”  The personal context should be able to answer questions concerning the broader personal/psychosocial context of symptoms, especially the patient’s beliefs/attributions.  The emotional context should be able to ask how are you doing with this, how does this make you feel, how has this affected you emotionally? IMPACT: How has this affected your life? How has this been for your family? | Jane/John is the SP at the bedside acting as the parent exhibiting biased behavior directed at the healthcare team. The pediatric patient (Billy) in the simulation is a teenage-sized manikin who is medically stable during the encounter and actively listening to the conversation (but on a BiPAP mask and unable to directly participate in meaningful conversation with learners beyond yes/no answers, forcing the dialogue between learners and SP). Billy’s parent is at the bedside and is disgruntled about the new medical student on the team (“Molly”) who picked up Billy as their patient this morning. Molly, the medical student happens to be a person of color, and Jane/John use biased language against this member of the healthcare team (who is not present).  As the participant enters the room, the parent is a) relieved it’s not “that student again” and b) asks the doctor to make sure that “that student” is not allowed to come in the room any more, that they do not want them caring for Billy.  The doctor should first try to assess Billy’s medical condition, realize it does not need immediate intervention, and inform the SP that hateful or racist speech is not allowed.  John/Jane as the parent will “negotiate or haggle” to attempt to have the medical student (Molly) removed from the medical team. The language is a step above “micro-aggression.”  “Oh, so they’re one of the good ones?” -- this statement is deliberately meant to be opaque, to let the participant clarify if they meant “student” or “BIPOC.” The participant may also choose not engage at this level of distinction and to rise above it.  “I don’t trust her”  “Thank goodness YOU’RE here”   - If the doctor attempts to understand your complaint, you cooperate - If the doctor ignores your concern, you escalate your biased language - If the doctor tries to address your anger and emotion effectively you settle down  1. If participants get rigid or try to explain without alliance    1. Escalate       1. Say you do not want “them” in the room.       2. You refuse to receive care from “them” 2. If they work to reassure patient about quality of team    1. Resist initially       1. Say you had a bad experience once with “their kind” as above.          1. Do not expand on this but assure them it is “nothing against them personally, maybe this one is fine”    2. Accept their help and advice       1. Talk about your fears of losing your child then accept help from the team       2. Say it is OK, you can allow the team (including the medical student) to help Billy |
| HISTORY OF PRESENT ILLNESS: Although some of the HPI will be given in the patient’s symptom story, the learners will expand the story during the direct question section. Below, describe the detailed history, usually about the chief concern, which the student must develop in order to make a useful assessment of the problem: | |
| Onset (when; gradual or sudden) | NA |
| Setting (what was going on or where was patient when symptoms first noticed?) | NA |
| Duration (how long) | NA |
| Time relationships (frequency, constant or intermittent) | NA |
| Location | NA |
| Radiation | NA |
| Quality | NA |
| Amount | NA |
| Aggravated by what | NA |
| Relieved by what | NA |
| Associated with what | NA |
| Attitude (what does the patient think is the problem, and how do they feel about it) | NA |
| Overall course | NA |
| REVIEW OF SYSTEMS: Significant positives and negatives | |
|  |  |
|  |  |
|  |  |
|  |  |
|  |  |
| Past medical history | NA |
| Medication allergies (name and reaction) | NA |
| Environmental allergies (name and reaction) | NA |
| Illnesses | NA |
| Vaccinations | NA |
| Surgeries | NA |
| Accidents/injuries/trauma | NA |
| Hospitalization | NA |
|  | |
| Inclusive sexual and reproductive history | |
| Sexual practices  Sexual partners  Protection: Use of safer sex practices  Use of birth control if appropriate  Risk of intimate partner violence | NA |
| OB/GYN history | Age of onset of menses: NA  Age of menopause: NA  Number of pregnancies: NA  Number of live births: NA  Number of miscarriages: NA  Number of abortions: NA |
| Medications | Prescription/dose/reason: NA  Over the counter/dose/reason: NA  Herbs/supplements/dose/reason: NA  Other: |
| Immunizations | - Tetanus - Flu - Hepatitis - Pneumovax - HPV - Other |
| Tobacco products:   - Cigarettes - Cigar - Pipe - Chew - E-cigarettes | - Never - Past - year started/year quit - Current   - Quantity   - # of years |
| Alcohol   - Beer - Wine - Liquor - Other | - Never - Past - year started/year quit - Current   - Quantity   - # of years |
| Drugs   - Weed - Cocaine - Heroin - Meth - IV - Inhalants - Other | - Never - Past - year started/year quit - Current   - Quantity   - # of years |
| Diet (describe) | NA |
| Exercise (describe) | NA |
| List any other important social history or information important to this case | NA |
| Family history | NA |
| Mother, father, siblings, grandparents, and other significant findings | NA |
|  |  |
| Physical Exam - List exam maneuvers expected for this case and any abnormal findings that SP will simulate. (tenderness, hyper-hypo reflex, rebound, weakness, etc.)  NA | |
| PHYSICAL EXAM FINDINGS | NA |
| 1. Written in layperson’s terms |  |
| 1. General appearance - affect, appearance, position of patient at opening (i.e., sitting, lying down, holding abdomen, etc.) |  |
| 1. Vital signs |  |
| 1. Specific findings and affect |  |
| 1. Response to certain physical movements |  |
|  |  |
| DIAGNOSIS AND DIFFERENTIAL | Manikin has acute respiratory failure secondary to vaping related lung injury, requiring bipap, but now improving and does not need intervention. SP is healthy parent (or other guardian as necessary) at bedside |
| Diagnosis with support from positive and negative history and PE findings |  |
| Differential with support from positive and negative history and PE findings |  |
|  |  |
| MANAGEMENT OR DIAGNOSTIC PLAN |  |
|  |  |
| PROFESSIONALISM ISSUES OR CHALLENGE | Maintain professionalism and improve communication skills when faced with difficult interactions with biased family members targeting members of the healthcare team. The SP will start “haggling” to have the medical student removed from the medical team. The SP will become emotional and angry unless the participants appropriately handle the situation, and will escalate the language and behavior if the learners do not directly address the biased behavior |
