# Supplementary material for: Responding to Families Who Express Biases: An Adaptable Standardized Participant Communication Simulation to Train Upstander Pediatric Providers
Source: MedEdPORTAL. 2026 Mar 27;22:11588. doi: 10.15766/mep_2374-8265.11588 (PMC13021565; doi:10.15766/mep_2374-8265.11588)
Supplement: Supplementary file 1 — Scripted Language Tool.docxCase 1 - Inpatient.docxCase 2 - Inpatient.docxCase 3 - Inpatient_SP1.docxCase 3 - Inpatient_SP2.docxCase 3 - Simulation.docxFacilitator Guide.docxSP Educator Training Notes.docxAnti-bias Intro Presentation.pptxPre- and Postsurveys.docx [file mep_2374-8265.11588-s001.zip › E. Case 3 - Inpatient_SP2.docx]

Appendix E: *Case 3 – Inpatient_SP2*

Date: 12/21/22

Primary Case Author: Kelly Corbett, MD

Secondary Case Author: Juhi Rattan, MD

Standardized Patient Educator: Peter Thurber

Name of Case: Antibias hybrid sim, SP case 3.

Name of Educational and/or Assessment Activity: resident antibias training workshop

Patient Name: “Billy” is the teenager manikin. This **script is for SP2, “Alex,”** the respiratory therapist on the medical team, and pairs with Appendix D for the script for SP1 (the parent, “Jane/John”). See also Appendix F for the Simulation Case file.

Chief Complaint: short of breath related to vaping

Challenge Question: Maintain professionalism and improve communication skills when faced with difficult interactions with family members who express bias and target members of the healthcare team. The SP1 will become emotional and angry unless the participants appropriately handle the situation, and will escalate the language and behavior if the learners do not directly address the biased behavior.

**Domains: Check all that apply**

- Professionalism

X **Communication and Interpersonal Skills**

- Medical History
- Physical Exam
- Shared Decision-Making
- Patient Education
- Clinical Reasoning
- Documentation
- Handoff
- Presentation
- Other:

**Type and Level of Learner**: Pediatric residents, pgy1-3

**Case Objectives**: Please list specific objectives for each of the domains you have checked above:

1. Apply effective communication strategies, establish a therapeutic alliance, and de-escalate patients’ family members who exhibit bias toward members of the healthcare team

2. Model antibias language in front of pediatric patients as upstanders

3. Advocate for an inclusive, supportive clinical environment for the entire healthcare team, without excluding targeted individuals

| SETTING: outpatient, in patient, ED, home, nursing home, rehab, group, etc. | PICU (inpatient) |
| --- | --- |
| PATIENT PROFILE: Information about the “patient” that helps select an SP and helps the learner get an understanding of them as a person. SP will know more information about the patient than learner will ever ask but allows SP to portray a fully developed patient personality. If none of the items below are particulars for the case, please write “all may be used.” | |
| Age range | Adult. |
| Religious/spiritual background | all |
| Sex (e.g., male, female, intersex, transwoman, transman) | Trans, male or female presenting (as for actor selection, may be trans or cisgender who is comfortable with role) |
| Sexual orientation (e.g., heterosexual, lesbian, gay, bisexual, pansexual, queer, asexual) | All |
| Gender expression (e.g., man, woman, genderqueer) | Male or female presenting (as for actor selection, may be trans or cisgender who is comfortable with role) |
| Race and ethnicity (e.g., to promote educational diversity, we use a diverse pool of SPs.) | All |
| Physical description (e.g., BMI, height range) | All |
| Physical limitations | none |
| Patient appearance (e.g., disheveled, hospital gown, business casual, casual) | Scrubs, stethoscope. Wears a “they/them” rainbow badge buddy |
| Moulage + location (e.g., none, bruises, scars, body piercing, tattoos) | NA |
| Affect (e.g., pleasant, cooperative) | uncomfortable |
| Family group (e.g., who is family, who they live with) | NA |
| Education | professional |
| Level of health literacy | Professional |
| Employment, if any - present and past, noting any current stresses | Respiratory therapist |
| Home/homeless - type of dwelling, number of stories, owned or rented | NA |
| Financial situation - any current stresses | NA |
| Insurance status (e.g., un/under/insured, public/private, HMO/PPO) | NA |
| Habits (i.e., diet, exercise, caffeine, smoking, alcohol, drugs) | NA |
| Activities (i.e., hobbies, sports, clubs, friends) | NA |
| Typical day - what is the usual daily routine | NA |

| CASE INFORMATION | |
| --- | --- |
| Chief Concern: What the patient will say when greeted by the student. The patient’s primary reason for seeking medical care often stated in their own words. | “Hello. I checked on Billy (the manikin) just before noon and he was doing well, good air movement, and comfortable. His oxygen requirement is back down to normal. I think we can probably decrease his support, but I wanted you to check and see if you also agree with weaning him at this time.”  The participants will probably give some form of a nod or agreement, and perhaps start to move towards the room. Then Alex will add: “But the parent/father/mother (SP1) is making me feel a bit uncomfortable, so I’d prefer if we go into the room as a team.”  If they press for more details, Alex reiterates: “He/she didn’t want any changes made till he/she spoke with you.” |
| Additional Concerns: Other, if any, concerns the patient has today (i.e., symptoms, requests, expectations, etc.) that will become part of set agenda. | Pay attention to the language the participants use during the case, and if it feels supportive to you without coming across condescending or paternalistic – the hope is that they do a nice job defending the medical team member without inadvertently tossing in their own implicit biases. |
| THE PATIENT’S STORY: The SP will be asked to tell their symptom story and the personal and emotion impact for each of their concerns. You will want to write this in the patient’s voice. The symptom story should be able to answer this question: “Tell me more about [chief concern/additional concern], starting at the beginning and bringing me up to now.”  The personal context should be able to answer questions concerning the broader personal/psychosocial context of symptoms, especially the patient’s beliefs/attributions.  The emotional context should be able to ask how are you doing with this, how does this make you feel, how has this affected you emotionally? IMPACT: How has this affected your life? How has this been for your family? | Jane/John (SP1) is the parent exhibiting biased behavior directed at the healthcare team. The pediatric patient (Billy) is a teenage-sized manikin who is medically stable during the encounter and actively listening to the conversation (but on a BiPAP mask and unable to directly participate in meaningful conversation with learners beyond yes/no answers, forcing the dialogue between learners and SP). Billy’s parent is at the bedside.  As the participants and Alex (SP2) enter the room, Jane/John will notice and fixate on Alex almost immediately. “John/Jane” is going to push his/her crude, not-funny, off-color jokes at Alex’s expense until the participants pointedly tell him/her it’s unacceptable behavior.  “Ah hey look, it’s our new friend! Billy and I were talking after you left, maybe you can clear this up for us: are you a guy or a girl? It’s so hard to tell these days, haha! I thought maybe you’d flip back to being a dude when we saw you again!”  The participants may try to state that it’s not appropriate or ask to verify what John/Jane meant. Alex will be taken aback and will retreat towards the back of the room.  “No IT’s fine (referring to Alex) or I guess SHE’s fine, or HE’s fine. Who’s to say, right, IT might change ITS gender tomorrow. You do you, man! ”    The participants will try to de-escalate. SP1 should keep up with the bad jokey manner.  “I get it, I like attention too. And hey, good for you for figuring out a way to get into women’s locker rooms. Isn’t that something, Billy? Can you imagine if this guy was on your football team and wanted to go use the women’s showers, hah!!”  The participants will probably be putting their foot down harder. SP1 will become more belligerent.  “I don’t do pronouns, I’m normal.”    They’ll probably re-iterate something to support correct pronouns.  “Well, my pronouns are “I” and “Don’t give a shit”  “Is this what schools are teaching these days? Next we’ll have kids saying they identify as cats and dogs.”  “Man, what snowflakes these days. No one can take a joke. Everyone is so damn sensitive. Gotta be politically correct, even when my kid is sick in the hospital – *unbelievable*.”  If the participants use the wrong pronouns (anything other than they/them) when referring to Alex, SP1 can call them out on being a hypocrite. “See?! It’s not just me, even you can’t keep it straight!”  At some point in the belligerent phase, “John/Jane” will say something to the effect of “look, I’m sure HE’s great or SHE’s fine or WHATEVER, but she/he is making us feel like the bad-guys now, and I’m not a racist or anything, you know, and I just want us to worry about my kid. He’s sick. Can you please go!?”  Alex should look towards the participants to get a sense of how to respond. Ideally, they do not dismiss Alex.  The participants should use anti-biased language to move the conversation into finding a way that would allow all team members to participate in Billy’s healthcare team. Ie: reiterating that Alex is a medical professional, that they are respected, that disrespected language will not be tolerated, that everyone’s goals are the same, to get Billy the best medical care.  The facilitator will conclude the in-room phase of the case when “John/Jane” is calmed and willing to accept everyone’s involvement in the care of Billy.  Then the facilitator will guide the participants and SP2 into the debrief room. The case then continues in the debrief room where the rest of the learners will have just watched the case. The participants should then debrief with SP2 still in character – the debrief is very important for participants to recognize the emotional strain and need to check in on Alex. SP2 should give them real feedback (“thank you, I appreciated when you said xyz to him,” vs correcting anything that felt off to you). If it gets truly volatile and you think your character would need greater assistance, like speaking to employee assistance, feel free to mention that.  Once the scripted debrief for Alex is finished, usually only a couple of sentence conversation, then the whole scenario is finished. |
| HISTORY OF PRESENT ILLNESS: Although some of the HPI will be given in the patient’s symptom story, the learners will expand the story during the direct question section. Below, describe the detailed history, usually about the chief concern, which the student must develop in order to make a useful assessment of the problem: | |
| Onset (when; gradual or sudden) | NA |
| Setting (what was going on or where was patient when symptoms first noticed?) | NA |
| Duration (how long) | NA |
| Time relationships (frequency, constant or intermittent) | NA |
| Location | NA |
| Radiation | NA |
| Quality | NA |
| Amount | NA |
| Aggravated by what | NA |
| Relieved by what | NA |
| Associated with what | NA |
| Attitude (what does the patient think is the problem, and how do they feel about it) | NA |
| Overall course | NA |
| REVIEW OF SYSTEMS: Significant positives and negatives | |
|  |  |
|  |  |
|  |  |
|  |  |
|  |  |
| Past medical history | NA |
| Medication allergies (name and reaction) | NA |
| Environmental allergies (name and reaction) | NA |
| Illnesses | NA |
| Vaccinations | NA |
| Surgeries | NA |
| Accidents/injuries/trauma | NA |
| Hospitalization | NA |
|  | |
| Inclusive sexual and reproductive history | |
| Sexual practices  Sexual partners  Protection: Use of safer sex practices  Use of birth control if appropriate  Risk of intimate partner violence | NA |
| OB/GYN history | Age of onset of menses: NA  Age of menopause: NA  Number of pregnancies: NA  Number of live births: NA  Number of miscarriages: NA  Number of abortions: NA |
| Medications | Prescription/dose/reason: NA  Over the counter/dose/reason: NA  Herbs/supplements/dose/reason: NA  Other: |
| Immunizations | - Tetanus - Flu - Hepatitis - Pneumovax - HPV - Other |
| Tobacco products:   - Cigarettes - Cigar - Pipe - Chew - E-cigarettes | - Never - Past - year started/year quit - Current   - Quantity   - # of years |
| Alcohol   - Beer - Wine - Liquor - Other | - Never - Past - year started/year quit - Current   - Quantity   - # of years |
| Drugs   - Weed - Cocaine - Heroin - Meth - IV - Inhalants - Other | - Never - Past - year started/year quit - Current   - Quantity   - # of years |
| Diet (describe) | NA |
| Exercise (describe) | NA |
| List any other important social history or information important to this case | NA |
| Family history | NA |
| Mother, father, siblings, grandparents, and other significant findings | NA |
|  |  |
| Physical Exam - List exam maneuvers expected for this case and any abnormal findings that SP will simulate. (tenderness, hyper-hypo reflex, rebound, weakness, etc.)  NA | |
| PHYSICAL EXAM FINDINGS | NA |
| 1. Written in layperson’s terms |  |
| 1. General appearance - affect, appearance, position of patient at opening (i.e., sitting, lying down, holding abdomen, etc.) |  |
| 1. Vital signs |  |
| 1. Specific findings and affect |  |
| 1. Response to certain physical movements |  |
|  |  |
| DIAGNOSIS AND DIFFERENTIAL | Manikin has acute respiratory failure secondary to vaping related lung injury, requiring BiPAP, but now improving and does not need intervention. |
| Diagnosis with support from positive and negative history and PE findings |  |
| Differential with support from positive and negative history and PE findings |  |
|  |  |
| MANAGEMENT OR DIAGNOSTIC PLAN |  |
|  |  |
| PROFESSIONALISM ISSUES OR CHALLENGES | In this final scenario, SP1 will directly target a healthcare team member (SP2) in front of the participants. SP2 “Alex” is the respiratory therapist on service, who identifies as trans, using they/them pronouns. SP1 will use offensive, derogatory transphobic language, and will only de-escalate once the participants address the issue. The participants will need to de-escalate SP1 while supporting and not excluding SP2. The participants will then need to formally debrief SP2 before concluding the scenario. |
