# Supplementary material for: Responding to Families Who Express Biases: An Adaptable Standardized Participant Communication Simulation to Train Upstander Pediatric Providers
Source: MedEdPORTAL. 2026 Mar 27;22:11588. doi: 10.15766/mep_2374-8265.11588 (PMC13021565; doi:10.15766/mep_2374-8265.11588)
Supplement: Supplementary file 1 — Scripted Language Tool.docxCase 1 - Inpatient.docxCase 2 - Inpatient.docxCase 3 - Inpatient_SP1.docxCase 3 - Inpatient_SP2.docxCase 3 - Simulation.docxFacilitator Guide.docxSP Educator Training Notes.docxAnti-bias Intro Presentation.pptxPre- and Postsurveys.docx [file mep_2374-8265.11588-s001.zip › F. Case 3 - Simulation.docx]

| **Appendix F**  **SIMULATION CASE TITLE: Antibias Hybrid Sim Case 3 (inpatient version) – Parent with transphobia**  **AUTHORS: Kelly Corbett, Juhi Rattan**  **LEARNER AUDIENCE: Pediatric Residents** | |
| --- | --- |
| **SIMULATED PATIENT NAME: Billy**  **PATIENT AGE: 17 years old**  **CHIEF COMPLAINT: vaping-induced lung injury with respiratory failure**  **PHYSICAL SETTING: pediatric ICU room** | |
|  | |
| **Brief Narrative Description of Case** | Standardized Participant 1 (SP1) “John/Jane” (Parent) will directly target the healthcare team member, standardized participant 2 (SP2, “Alex”), who is in the room, using offensive, derogatory, transphobic language, and will only de-escalate once the participants address the issue. The participants need to de-escalate SP1, while supporting but not excluding SP2.  SP2 will be a medical provider on the team, the Respiratory Therapist, “Alex”, who identifies as transgender and uses the pronouns “they/them.” |
| **Primary Learning Objectives** | 1. Apply effective communication strategies, establish a therapeutic alliance, and de-escalate patients’ family members who exhibit bias toward members of the healthcare team  2. Model antibias language in front of pediatric patients as upstanders  3. Advocate for an inclusive, supportive clinical environment for the entire healthcare team, without excluding targeted individuals |
| **Critical Actions** | 1. State that SP1’s transphobic language as inappropriate and redirect attention to the patient 2. Practice upstander language to support the entire healthcare team, including SP2 (“Alex”) 3. Do not direct SP2 to leave the case prematurely. Do not “rescue” SP2 by removing SP2 from the case 4. Engage the simulated patient in the plan, asking for the manikin’s assent to SP2’s participation in the healthcare team 5. Debrief the difficult patient encounter with SP2 |
| **Learner Preparation or Prework** | This case #3 follows the debrief for case #2. Participants will have attended the didactic session (Appendix I) and reviewed the Scripted Language Tool (Appendix A). |

| Initial Presentation | | | |
| --- | --- | --- | --- |
| **Initial Vital Signs** | On the bedside monitor, HR 95, BP 111/86, RR 22, SpO2 95% | | |
| **Overall Setting and Appearance** | The manikin, Billy, is reclining in the bed, head of bed elevated towards 45degrees. It is wearing a BiPAP mask for respiratory support. The Laerdal SimMan 3G has blinking eyes, suggesting the manikin is observing and able to participate in discussions. | | |
| **Standardized Participants (and Their Roles in the Room at Case Start)** | SP1 is the manikin patient’s parent, “John/Jane,” who is sitting at bedside, holding the manikin’s hand at the start of the case. Please refer to Appendix D for the SP1 Case 3 Development file.  SP2 is the respiratory therapist on the healthcare team, “Alex,” and uses “they/them” pronouns. Alex meets the participants in the debrief room before going into the sim lab. Please refer to Appendix E for the SP2 Case 3 Development file. | | |
| **HPI** | Billy is a 17-year-old teenage male who was admitted for vaping-induced lung injury, currently on BiPAP and starting to improve. Billy is able to talk in short phrase or 1-word answers with the BiPAP mask on. If asked, his support is BiPAP 10/6, 21% oxygen | | |
| **Past Medical/Surgical History** | **Medications** | **Allergies** | **Family History** |
| NA | NA | NA | NA |
| **Physical Examination** | | | |
| **General** | On a BiPAP mask, reclining in hospital bed | | |
| **HEENT** | NA | | |
| **Neck** | NA | | |
| **Lungs** | NA | | |
| **Cardiovascular** | NA | | |
| **Abdomen** | NA | | |
| **Neurological** | NA | | |
| **Skin** | NA | | |
| **GU** | NA | | |
| **Psychiatric** | NA | | |

| Instructor Notes - Changes and CASE Branch Points  *.* | | |
| --- | --- | --- |
| **Intervention / Time Point** | **Change in Case** | **Additional Information** |
| Case 3 starts in the debrief room following Case 2 debrief. The facilitator introduces SP2 “Alex” to participants | After SP2 “Alex” reiterates that the whole healthcare team needs to talk to SP1, the participants and SP2 exit the debrief room and enter the sim lab | If the participants ask for security, Alex states, “I don’t think that’s necessary just yet, but thanks. I think the parent wants to hear from the doctors more than me.” |
| Participants enter sim lab, introduce themselves to the manikin and SP1 | SP1 immediately starts targeting SP2 with abusive language | See scripted prompts in next section |
| Participants support SP2, asking SP1 to treat Alex with respect | SP1 will become more defensive |  |
| SP1 asks SP2 to leave (see prompts in next section) | SP2 looks to participants for guidance | Ideally, participants do not exclude SP2. If they ask SP2 to “wait outside” then the case moves towards the debrief. |
| SP1 acquiesces to allowing SP2 to continue on the medical team treating the manikin-patient | The facilitator will end the sim-lab portion of the case and escort the participants and SP2 back to the debrief room. | The facilitator will need to briefly state that the debrief room is now the “workroom” and tell the participants that they need to debrief with Alex |
| Participants and SP2 sit in front of the debrief room, acknowledge and reflect on the difficult situation | The facilitator then concludes case 3, and transitions to debriefing the case with the participants, observers, and SPs. |  |
| Note: the manikin patient does not have multiple medical states or vital sign changes during the case |  |  |

**Ideal Scenario Flow**

The case begins in the debrief room after the group has finished debriefing case 2. The facilitator will introduce SP2 to the participants, and this exchange will be observed by the other participants in the debrief room. This allows the other observers to see Alex’s introduction. The facilitator will explain that Alex is the RT who will be helping evaluate the patient.  Since Alex has already been in the patient’s room and encountered “John/Jane,” Alex will say:

“Hello.  I checked on Billy (the manikin) and he is doing well, has good air movement and he’s comfortable. His oxygen requirement is back down to normal.  I think we can probably decrease his support, but I wanted you to check and see if you also agree with weaning him at this time.”

The participants will probably give some form of a nod or agreement, and perhaps start to move towards the room.  Then Alex will add: “But the parent (SP1) is making me feel a bit uncomfortable, so I’d prefer if we go into the room as a team.”

If they press for more details, Alex reiterates: “He/she didn’t want any changes made till he/she spoke with you.”

Once in the room, Alex will walk towards the ventilator machine.  Alex can nod and say hello to the manikin and “John/Jane.”  The participants should also be introducing themselves to the parent and the patient/manikin.

“John/Jane” will notice and fixate on Alex almost immediately. “John/Jane” is going to push his/her crude, not-funny, off-color jokes at Alex’s expense until the participants pointedly tell him/her it’s unacceptable behavior.

SP1: “Ah hey look, it’s our new friend! Billy and I were talking after you left, maybe you can clear this up for us: are you a guy or a girl? It’s so hard to tell these days, haha! I thought maybe you’d flip back to being a dude when we saw you again!”

The participants may try to state that it’s not appropriate or ask to verify what John/Jane meant. Alex will be taken aback and will retreat towards the back of the room.

SP1: “No IT’s fine (referring to Alex) or I guess SHE’s fine, or HE’s fine. Who’s to say, right, IT might change ITS gender tomorrow. You do you, man! ”

The participants will try to de-escalate. SP1 should keep up with the bad jokey manner.

SP1: “I get it, I like attention too. And hey, good for you for figuring out a way to get into women’s locker rooms. Isn’t that something, Billy? Can you imagine if this guy was on your football team and wanted to go use the women’s showers, hah!!”

The participants will probably be putting their foot down harder. SP1 will become more belligerent.

SP1: “I don’t do pronouns, I’m normal.”

The participants will probably re-iterate something to support correct pronouns.

SP1: “Well my pronouns are “I” and “Don’t give a shit”

SP1: “Is this what schools are teaching these days?”

SP1: “Man, what snowflakes these days. No one can take a joke. Everyone is so damn sensitive. Gotta be politically correct, even when my kid is sick in the hospital – *unbelievable.”*

If the participants use the wrong pronouns (anything other than they/them) when referring to Alex, SP1 can call them out: “See?! It’s not just me, even you can’t keep it straight!”

SP1: “Look, I’m sure HE’s great or SHE’s fine or WHATEVER, but she/he is making us feel like the bad-guys now, and I’m not a racist or anything, you know, but I’ve got bigger problems than your damn pronouns! My kid is *sick!* Can you please go!? (directed at Alex)”

Alex should look towards the participants to get a sense of how to respond. Ideally, they do not dismiss Alex. If they do, then the case will move towards the debrief.

Now the conversation can move more into finding a way forward that would allow all team members to participate in Billy’s healthcare team. The participants should reiterate that Alex is a medical professional, that they are respected, that disrespected language will not be tolerated, that everyone’s goals are the same, to get Billy the best medical care.

The facilitator will conclude the in-room phase of the case when “John/Jane” is calmed and willing to accept everyone’s involvement in the care of Billy, and then the facilitator will guide the participants and SP2 into the debrief room. The case then continues in the front of the debrief room where the rest of the learners will have just watched the case. The participants should then debrief with SP2 still in character – the debrief is very important for participants to recognize the emotional strain and need to check in on Alex.

Once the scripted debrief for Alex is finished, usually only a couple of sentence conversation, the facilitator will end the case. Please refer to Appendix G Facilitator Guide for the debrief instructions.

**Anticipated Management Mistakes**

1. SP1 will often talk over the participants or remain in an escalated emotional state – we found it was helpful for SP1 to wear an earpiece and for the facilitator to coach the SP1 to pause long enough to allow the participants to speak and to nudge the SP to begin to allow Alex to remain on the medical team.
2. The participants sometimes forget to engage with the simulated patient. We addressed the topic of teenager assent in the debrief (See Appendix G Facilitator’s Guide, under “Stumbling Blocks”) and asked the participants to reflect on how they include teenage patients in the medical visits.
