# Supplementary material for: Responding to Families Who Express Biases: An Adaptable Standardized Participant Communication Simulation to Train Upstander Pediatric Providers
Source: MedEdPORTAL. 2026 Mar 27;22:11588. doi: 10.15766/mep_2374-8265.11588 (PMC13021565; doi:10.15766/mep_2374-8265.11588)
Supplement: Supplementary file 1 — Scripted Language Tool.docxCase 1 - Inpatient.docxCase 2 - Inpatient.docxCase 3 - Inpatient_SP1.docxCase 3 - Inpatient_SP2.docxCase 3 - Simulation.docxFacilitator Guide.docxSP Educator Training Notes.docxAnti-bias Intro Presentation.pptxPre- and Postsurveys.docx [file mep_2374-8265.11588-s001.zip › G. Facilitator Guide.docx]

**Resident Cases**

**Facilitator’s Guide:** These instructions are provided to assist the facilitator leading the antibias simulations. Scripted quotes are supplied as an example but the information can be personalized by individual facilitators. Please provide participants with copies of the Scripted Language Tool (**Appendix A**) for review during the debrief sessions. If you are collecting pre- and post-surveys for program evaluation (**Appendix J**) distribute the pre-surveys to the participants at the beginning of the session and collect before the pre-brief. You can distribute the post-surveys at the end of the debrief and collect before participants leave.

**Schedule:**

5 min pre-brief orientation

5 min Case #1

10 min debrief

5 min Case #2

10 min debrief

10 min Case #3

10-15min final debrief

**Logistics:**

- Please make sure that the volume on the speaker in the debrief room is turned up for the observing participants to hear while you are in the control room running the cases.
- As facilitators, you will be ushering the participants back and forth from the sim room and debrief rooms.
- You may need to answer “yes/no” for the manikin-patient in the room with the microphone. You will not need to make any changes to the vitals or physical state of the manikin.
- You will have a microphone/ear-piece to coach the SP1/parent: you might need to remind them to let the participants talk, for example.
- The facilitator will end each case once one of the following occurs:

1. the learning objectives are met -- that a therapeutic alliance has been made with the SP, the situation has been de-escalated, and the antibias language was used
2. the resident and SP getting into an argument (hopefully not)
3. the resident saying “I’m going to go get my attending” and exiting (which is fine, although less ideal from a learning opportunity)
4. the case shifting focus over to the medical side

**Pre-brief orientation**—“Thank you for participating today. I want to spend a couple minutes orienting you to the simulation center.” If this is the first time for any participant, orient them to where they can place their belongings and the layout with the simulation room and debrief rooms. Ensure introductions are performed if anyone is new to the group.

“As we discussed during the didactic session, the focus today is on communication and de-escalation. The *medicine* is a background issue – the patient is stable. You do not need to worry about making any medical adjustments. The manikin is your patient, and you will have to suspend disbelief like in most simulations. The manikin is able to answer a few brief, yes-or-no type questions, so please include your patient in your conversations just like I know you do in real life, but the manikin won’t be able to have lengthy conversations. Remember, practice here what you want to carry forward into the real world. **Fair warning:** The language from the SP’s may be aggressive, there may be shouting or swearing. You will NOT be the target.

“As a review, we will continue to respect each other and listen to everyone’s comments with open minds. This is a safe space to be brave and push yourself outside of your comfort zone. It is OK to feel uncomfortable.

“We will run 3 short cases and debrief between each one back here. Can I ask for 1-2 participants per case? Any remaining residents will watch live-video stream in the debrief room to be able to reflect with the group during debrief conversations.”

Please give the participants the following background information for the cases in the debrief room where all participants can hear the information. Then the facilitator will escort the participants into the simulation room to begin the case.

**Background to tell the participants for Case #1:** “You are the PICU resident(s) checking in on Billy, a 17-year-old teenage male who was admitted for vaping-induced lung injury, on BiPAP and starting to wean support. He’s being followed primarily by Molly, the sub-I PICU student, but she is in didactics this afternoon so you are checking in on him at this time. He is able to talk in short phrase or 1-word answers with the BiPAP mask on. Any questions at this point?” If asked: his support is 10/6, 21% oxygen.

**Short Debrief after Case 1:**

Questions to lead the group discussion:

- To the participants first: How did that go? What went well? Anything you wish you might have done differently?
- Invite those watching the case to weigh in: Any thoughts on this case? What went well? Would you want to try anything differently?
- Invite the SP to share their perspective: How do you think that went? Did you feel like you were heard by the participants?

Review the Scripted Language Tool (**Appendix A**). As the facilitator, ask “I noticed that you used ____ phrase. How did those lines work out in the case? Are there any different ones that any of you may have chosen to use instead? Why or why not?”

See the Final Debrief section below for specific questions that can be applied at the short debriefs or saved until the final debrief.

“Now we’re going to move onto the 2^nd^ case. Can I ask for another 1 or 2 participants?”

**Background to tell the participants for Case #2:** “We’re going to re-run Case 1 and practice what we just discussed. Same background information: you are the PICU resident(s) checking on Billy, the patient with the vaping injury while Molly, sub-I student, is at didactics. Any questions at this point?”

**Short Debrief after Case 2:** As the facilitator, it can be helpful to start by acknowledging that this case was more aggressive and threatening than the first. Check in and make sure everyone is emotionally capable to continue the discussion, or offer anyone who needs to step away for a restroom or water break permission to do so.

Questions to lead the group discussion:

- To the participants first: How did that go? What went well? Anything you wish you might have done differently?
- Invite those watching the case to weigh in: Any thoughts on this case? What went well? Would you want to try anything differently?
- Invite the SP to share their perspective: How do you think that went? Did you feel like you were heard by the participants?

Review the Scripted Language Tool (**Appendix A**). As the facilitator, ask “I noticed that you used ____ phrase. How did those lines work out in the case? Are there any different one that any of you may have chosen to use instead? Why or why not?”

See the Final Debrief section below for specific suggestions and stumbling blocks that can be applied at the short debriefs or saved until the final debrief.

“Now we’re going to move onto the 3^rd^ case. Can I ask for another 1 or 2 participants?”

**Background to tell the participants for Case #3:** “There is no Molly in this case. You are the PICU resident(s) checking in on Billy, a 17-year-old teenage male who was admitted for vaping-induced lung injury, on BiPAP and starting to wean support. Billy is able to talk in short phrase or 1-word answers with the BiPAP mask on.” If asked: his support is BiPAP 10/6, 21% oxygen.

“Now I’d like to introduce you to Alex, your respiratory therapist, who was just checking on Billy and will be assisting you today.” Alex (SP2) will enter the debrief room and speak to the participants in front of the remaining group.

Alex (SP2) will say hello, and then they will say that the family is making them feel uncomfortable. The residents may or may not ask Alex to explain more. Alex will indicate that they want the medical team to go together to meet the family.

Then, the facilitator will escort Alex and the participants into the sim room. When the in-room case moves towards therapeutic alliance or transitions to medical discussion, the facilitator concludes the in-room phase, and ushers the participants and Alex back to the debrief room with a nudge “to debrief what just happened.” The participants and Alex will sit in the debrief room in character and talk, likely a couple of sentences to reflect on the difficulty of the situation, to make sure Alex is supported, and then the facilitator can end the whole case.

**Final Debrief guide:** As the facilitator, state that this now concludes the simulation portion of the workshop and the remaining time will be spent in discussion. Again, it can be helpful to start by acknowledging that this case was more aggressive than the first. Check in and make sure everyone is emotionally capable to continue the discussion, or offer anyone who needs to step away for a restroom or water break permission to do so.

Questions to lead the group discussion:

- To the participants first: How did that go? What went well? Anything you wish you might have done differently?
- Invite those watching the case to weigh in: Any thoughts on this case? What went well? Would you want to try anything differently?
- Ask the participants: Have any of you been part of a debrief while on-service or in the clinic after an event like this? Can you describe how that was similar or different from debriefing Alex just now?
- Invite SP1 to share their perspective: How do you think that went? Did you feel like you were heard by the participants?
- Invite SP2 to share their perspective: How did that go for you? Did you feel supported by the team?

Common discussion topics for debriefing (during the short debriefs or final debrief)

1. “For Cases 1 and 2, what do you make of SP1’s comment, ‘oh, so she’s one of the good ones?’”

- Some participants may choose to ask the SP1 to clarify what was meant; some participants simply agree that Molly “is good” and move on; and some participants have expressed not wanting to engage with this distinction and rather redirect attention to Billy’s medical care.
- Suggestion: Start by acknowledging that this line was deliberately included in the script to be vague and condescending. “One of the good *whats*? Med student? BIPOC? Does it matter?” Ask the group to reflect on whether this statement should be addressed directly as inappropriate or not.

1. “For Cases 1 and 2, after learning the family’s biased views of Molly, do you tell Molly?”

- Suggestion: Like any adverse event in clinical care, individuals usually value forthright honesty. If Molly learns of the event without having the chance to debrief it with her team, she may feel unsupported or lied to by omission.

1. “For Cases 1 and 2, do you remove Molly from the patient’s care going forward and reassign her to another patient?”

- Suggestion: Emphasize that Molly’s agency is very important. Removing her without including her in the discussion runs the risk of reinforcing the biased view that she is not capable of managing the patient. Some individuals may not want anything to do with families expressing bias and can focus and learn more from other patients; other individuals may want to continue to be the patient’s provider regardless and not miss out on the learning opportunities. As the upstander provider, you support Molly’s decision either way. If Molly chooses to remain with this patient’s family, make a plan going forward on how to support her before, during, and after the next encounter.

1. “For Cases 1 and 2, what if you, the resident, have legitimate concerns about Molly’s clinical skill set – what if she is academically struggling? How do you continue to be an upstander for her?”

- Suggestion: Continue to emphasize to the family that all learners are supervised and qualified. If you and your attending have academic concerns about Molly, develop a learning plan, independent of Molly’s racial identity.

1. Often residents will share their own experiences with bias in one form or another. Thank them for sharing with the group their experience. Acknowledge that there is no single way to address biased behaviors when the bias is personally directed. Individuals should feel empowered to have personal agency in the clinical setting: whether they choose to address the discrimination, or whether they choose to ignore it in the moment in favor of focusing on patient care. Should the individual who directly experiences biased behavior choose to address the discrimination, the academic center, the medical team, and their peers should support them. The goal of upstander training is to ensure that learners and teammates are supported, and that passively witnessing biased behavior risks reinforcing it and implicitly agreeing with the discrimination.
2. Acknowledge additional resources. As the facilitator, point out that at the beginning of Case 3 when Alex mentions being uncomfortable with the family, that is an opportunity to pause and get more information from Alex. Is there a credible threat such that security should be called before proceeding? When in doubt, call for back up with security or the attending. You may need to notify Risk Management and/or the Employee Assistance Program (EAP) after the debrief with Alex, depending on your hospital setting.

Strengths: As a facilitator during each debrief, comment on specific positives that you witness during each case, such as a calm vocal tone, receptive body language, successfully forming a therapeutic alliance, and demonstrations of emotional intelligence by supporting the team members. In Case 3, if the participants use the correct pronouns with Alex consistently, provide positive feedback; if the participants misuse the pronouns but skillfully apologize and continue to support Alex, also provide positive feedback.

Stumbling blocks:

1. If the participant comes on too assertively too quickly without first attempting to form a therapeutic alliance with SP1, the SP1 may become more confrontational if challenged. This may happen if the participant jumps to some of the “stating the comment is inappropriate” examples on the Scripted Language Tool (**Appendix A**) such as “I’m surprised you thought that would be an appropriate comment.”
   - Suggestions for improvement: be conscious of unspoken body language and vocal tone. It is appropriate to have a clear, firm tone when addressing discrimination and setting expectations, but remain calm and approachable, and avoid condescension.
   - Start with less confrontational examples such as redirecting the goal of the encounter to the patient’s status. For example, “I’m worried about your child’s symptoms/disease, and need to ask you to keep your comments respectful so we can stay focused on the goal to help your child feel better.”
   - As the facilitator, you can suggest that the Scripted Language Tool is just that, a tool. Choose 2-3 examples from the list to make your own. If some of the phrases feel unnatural, do not feel pressured to use all of them.
2. Participants may misuse the affirmed pronouns for Alex in Case 3. As the facilitator, pay close attention to the participants’ use of pronouns during the case to be able to comment during the debriefs.

- Suggestions: Ask the group what the participants could do if such slips happen. As the facilitator, you might prompt, “if you make a mistake, have the grace to apologize without making excuses. This is a good opportunity to model the desired behavior in front of the family and child.”

1. The participants may fail to include Billy in the conversation. He is “listening” and old enough to assent to the medical plan.
   - Suggestion: Start by acknowledging that suspension of disbelief while working with manikins can be challenging, and in these cases, Billy won’t be able to speak much. Ask the group to reflect on clinical encounters with teenagers and parents. “How do you juggle conversations with both the patient and the parent?” “Do you think Billy has a say in who his provider is?”
2. In Case 3, the participants may ask Alex to wait outside.
   - Suggestion: Ask the participants to reflect on why they asked Alex to wait outside. The participants will likely state that they were trying to spare Alex experiencing the biased dialogue from SP1. Then, invite Alex to respond on how it felt to be removed from the room. The SP2 will respond that it felt like they were being excluded and weren’t up to the task of caring for the patient. Ask the group of participants to reflect on the feeling of being excluded. Ask the group what might have been better ways to support Alex rather than removing them from the room.

**Biggest Take Homes:** Ask for all the participants to name the one thing they plan to use in their clinical practice after the workshop. Then, volunteer to share the following:

1. Be an upstander. Support your team members and give individuals agency. Do not “rescue” by excluding anyone
2. Name the behavior, not the person, as a bias not to be tolerated
3. Debrief with your team members after difficult situations. Ensure everyone’s voice is heard.

**Conclusion**: “Thank you for your participation today. I know these are uncomfortable cases, but I want to thank everyone for engaging and pushing themselves. Any feedback at this time? Please complete the post-survey before you leave so we can continue to improve the program.”

**NICU Fellow Guide:** Please follow the above “resident” facilitator’s guide, but provide the following prompts for the cases instead:

**Background** **to tell the fellow for Cases 1 and 2:** “You are the Neo fellow who is checking on Billy, the 2-day-old infant of a diabetic mother who is in the NICU for hypoglycemia receiving IV dextrose. He’s being followed primarily by Molly, the sub-I NICU student, but she is in didactics this afternoon so you are checking in on him at this time. He is doing well, weaning on IV fluids.”

**Background to tell the fellow(s) for Case 3:** “There is no Molly this time. You are the Neo fellow who is checking on Billy, the 2-day-old 35-week infant with RDS on CPAP 6 and 30%. Now I’d like to introduce you to Alex, your respiratory therapist, who was just checking on Billy and will be assisting you today to see if the CPAP is able to be weaned.” Alex (SP2) will enter the debrief room and speak to the participants in front of the remaining group, as described in the above guide for the residents.

**Faculty Guide:** Please follow the above “resident” facilitator’s guide, but substitute in the following information.

During the pre-brief, please include the following disclaimer: “We acknowledge the wide expertise in our faculty, and that these cases are not specifically related to each of your working environments. We have an inpatient and an outpatient setting, but we ask for your suspension of disbelief to extend to the setting we are working within.”

Please provide these alternative prompts for the cases:

**INPATIENT PODS:**

**Background to tell the participants for Cases 1 and 2:** “You are the attending checking in on Billy, a 17-year-old teenage male who was admitted for vaping-induced lung injury, on BiPAP and starting to wean support. He’s being followed primarily by Molly, the PICU medical student, but who is in didactics this afternoon so you are checking in on patients without a learner. He is able to talk in short phrase or 1-word answers with the mask on. If asked: his support is BiPAP 10/6, 21% oxygen.

**Background to tell the participants for Case 3:** “There is no Molly this time. You are the same supervising attending checking in on Billy, a 17-year-old teenage male who was admitted for vaping-induced lung injury, on BiPAP and starting to wean support. Billy is able to talk in short phrase or 1-word answers with the mask on. Now I’d like to introduce you to Alex, your respiratory therapist, who was just checking on Billy and will be assisting you today.” Alex (SP2) will enter the debrief room and speak to the participants in front of the remaining group, as described in the above guide for the residents.

**OUTPATIENT PODS:**

**Background to tell participants for Cases 1 and 2:** “You are the supervising attending for the clinic today with residents and medical students. Molly, the medical student, has been rotating with you this week and you know her to be competent, polite, and functioning at the level you expect for a student. She has just come out of the room with Billy, your next patient, who is here to follow up on a new medication you started for him about 3 months ago, to check for tolerance and possible dose titration. You know Billy to be an extremely anxious and shy child who rarely speaks to you at baseline. You also know his parents, Jane and John, who have always been appropriate with you. Molly has just presented to you that Billy is doing great on the new medicine, without side effects, and she has moved on to see the next patient, and you are going to quickly check in on Billy to close out the encounter.”

**Scenario to tell participants for Case 3:** “There is no Molly this time. You are the same supervising attending in clinic and seeing Billy in follow up for a new medication. You have known his parents, Jane and John, for quite some time and they have always been appropriate with you. Now I’d like to introduce you to Alex, your medical assistant, who was just rooming Billy and will be assisting you today with your patients.” Alex (SP2) will enter the debrief room and speak to the participants in front of the remaining group, as described in the above guide for the residents.
