# Supplementary material for: Responding to Families Who Express Biases: An Adaptable Standardized Participant Communication Simulation to Train Upstander Pediatric Providers
Source: MedEdPORTAL. 2026 Mar 27;22:11588. doi: 10.15766/mep_2374-8265.11588 (PMC13021565; doi:10.15766/mep_2374-8265.11588)
Supplement: Supplementary file 1 — Scripted Language Tool.docxCase 1 - Inpatient.docxCase 2 - Inpatient.docxCase 3 - Inpatient_SP1.docxCase 3 - Inpatient_SP2.docxCase 3 - Simulation.docxFacilitator Guide.docxSP Educator Training Notes.docxAnti-bias Intro Presentation.pptxPre- and Postsurveys.docx [file mep_2374-8265.11588-s001.zip › H. SP Educator Training Notes.docx]

Standardized Participant (SP) Educator Notes:

These instructions are provided to assist in training the SPs for the antibias cases 1-3.

Please provide an overview of the purpose of the workshop and the progressive nature of the 3 cases. The first 2 cases are written for the parent (SP1) of a pediatric manikin patient who expresses race-based discrimination against a medical student (not present at the time) and the 3^rd^ case is written for the parent (SP1) to express transphobic language against a present member of the medical team (SP2).

1. Share the Scripted Language Tool with the SPs (Appendix A). The participants are practicing these phrases, and the SPs found it helpful to know what phrases to listen for.
2. SP1 tends to monologue. Re-iterate that they need to let the participants get a word in to try to de-escalate.
3. The tone for SP1 in case 1 can be thought of as “haggling” to try to remove the medical student from the medical team because of her race. This escalates in case 2, starts out “loudly complaining” and can reach the point of shouting if the participant struggles to make a therapeutic alliance and de-escalate the parent (SP1).
4. SP2 actors tended to want to understand a bit about what a respiratory therapist does and their role on the healthcare team. The following information was shared: “Respiratory therapists are part of our multidisciplinary team in the hospital, responsible for helping patients with breathing treatments (like nebulizers or cystic fibrosis treatments) and managing our ventilator equipment (breathing machines like CPAP or ventilators for respiratory failure). Physicians work very closely with RTs, no different than working closely with a patient’s bedside nurse.”
