# Supplementary material for: Responding to Families Who Express Biases: An Adaptable Standardized Participant Communication Simulation to Train Upstander Pediatric Providers
Source: MedEdPORTAL. 2026 Mar 27;22:11588. doi: 10.15766/mep_2374-8265.11588 (PMC13021565; doi:10.15766/mep_2374-8265.11588)
Supplement: Supplementary file 1 — Scripted Language Tool.docxCase 1 - Inpatient.docxCase 2 - Inpatient.docxCase 3 - Inpatient_SP1.docxCase 3 - Inpatient_SP2.docxCase 3 - Simulation.docxFacilitator Guide.docxSP Educator Training Notes.docxAnti-bias Intro Presentation.pptxPre- and Postsurveys.docx [file mep_2374-8265.11588-s001.zip › I. Antibias Intro Presentation.pptx]

## Slide 1
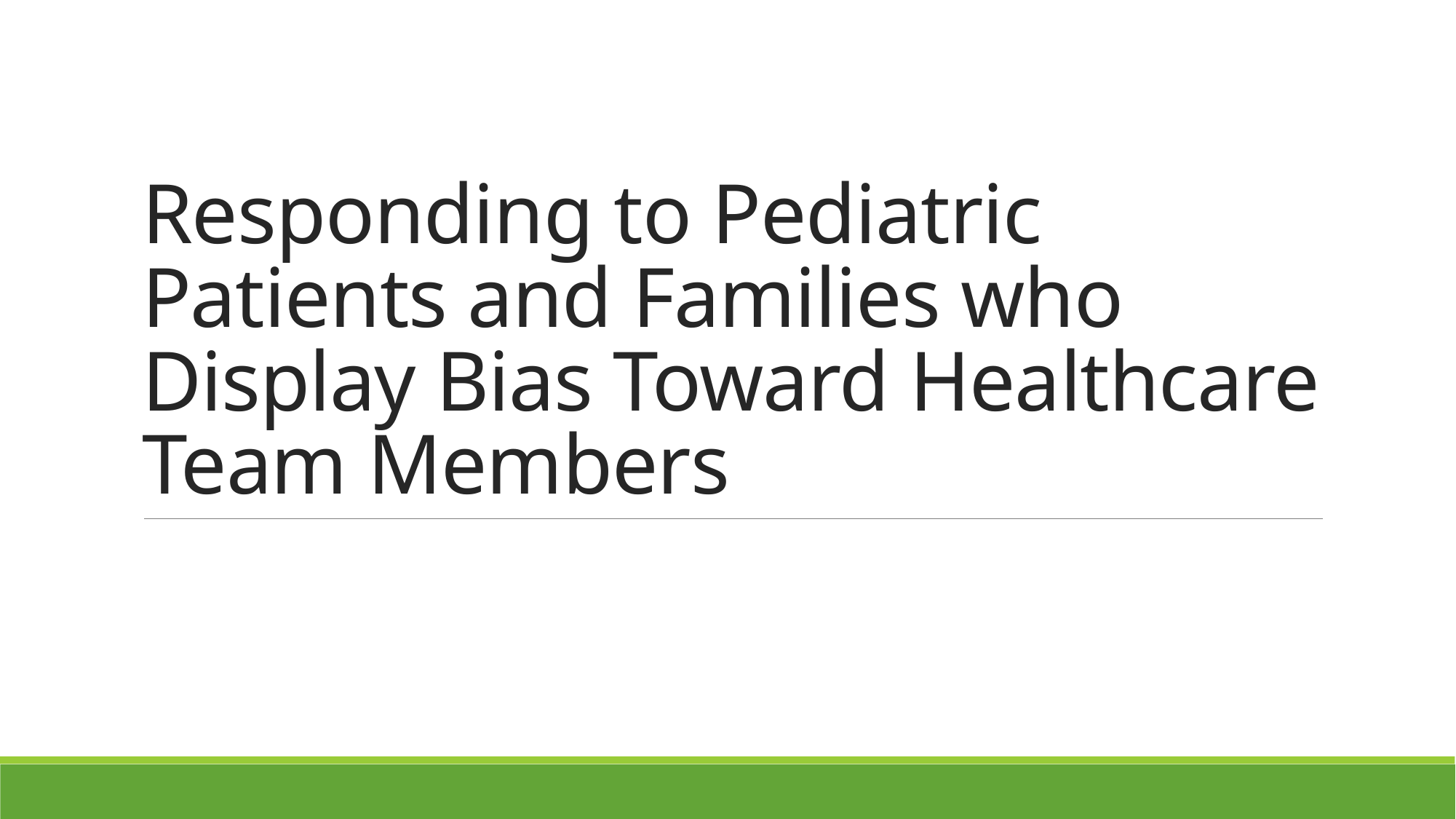

# Responding to Pediatric Patients and Families who Display Bias Toward Healthcare Team Members

## Slide 2
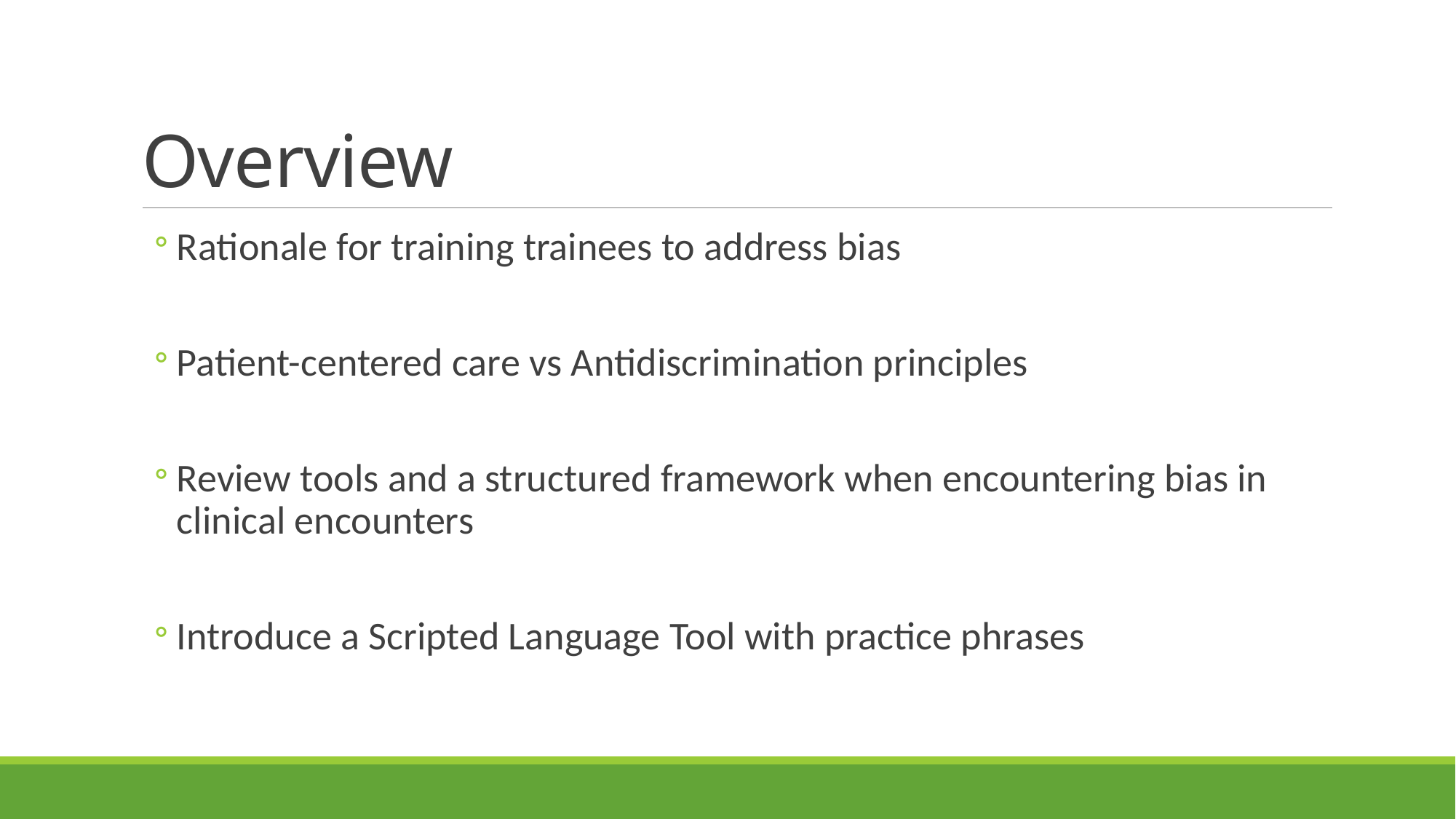

# Overview
Rationale for training trainees to address bias
Patient-centered care vs Antidiscrimination principles
Review tools and a structured framework when encountering bias in clinical encounters
Introduce a Scripted Language Tool with practice phrases

## Slide 3
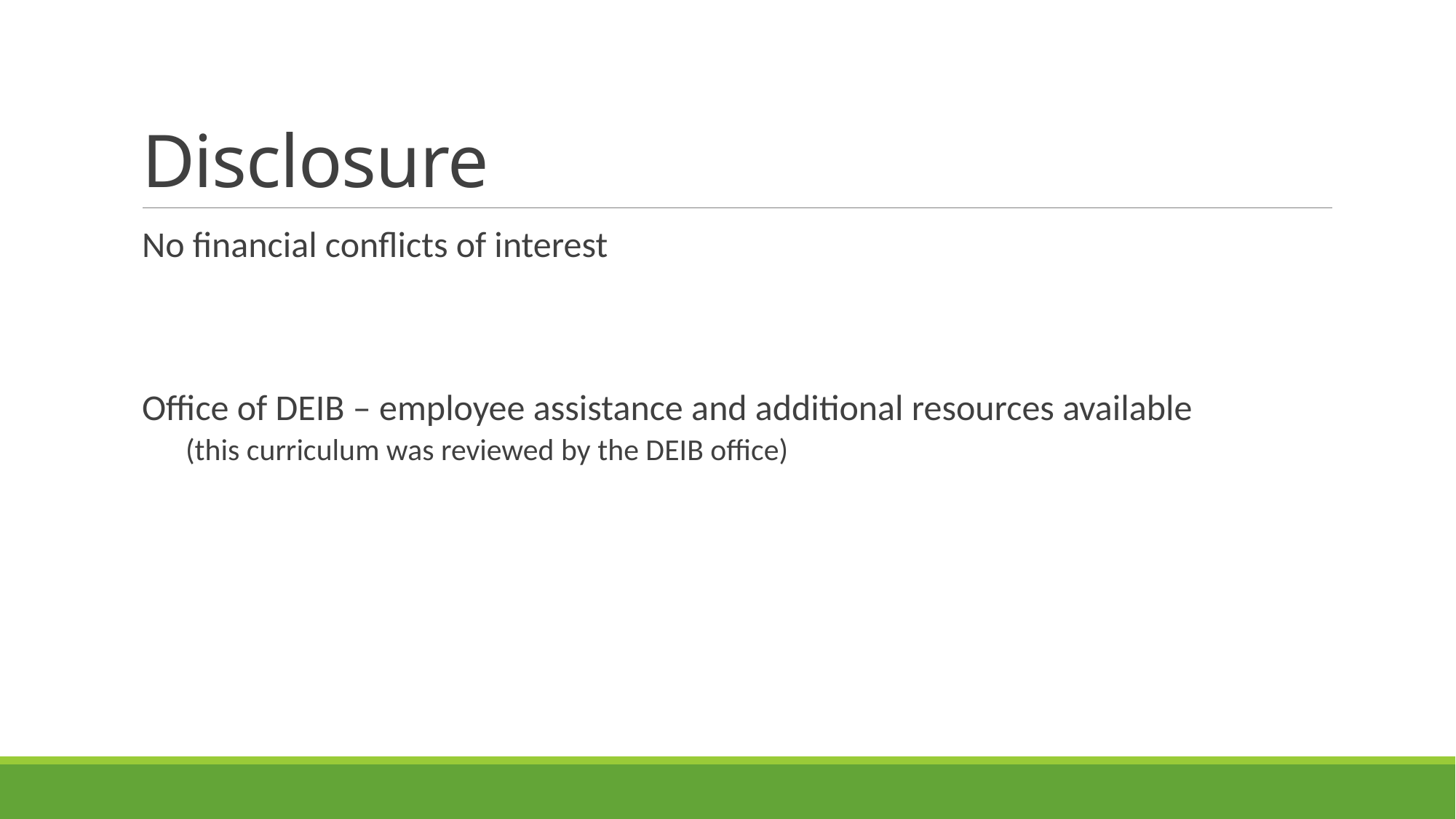

# Disclosure
No financial conflicts of interest
Office of DEIB – employee assistance and additional resources available
(this curriculum was reviewed by the DEIB office)

## Slide 4
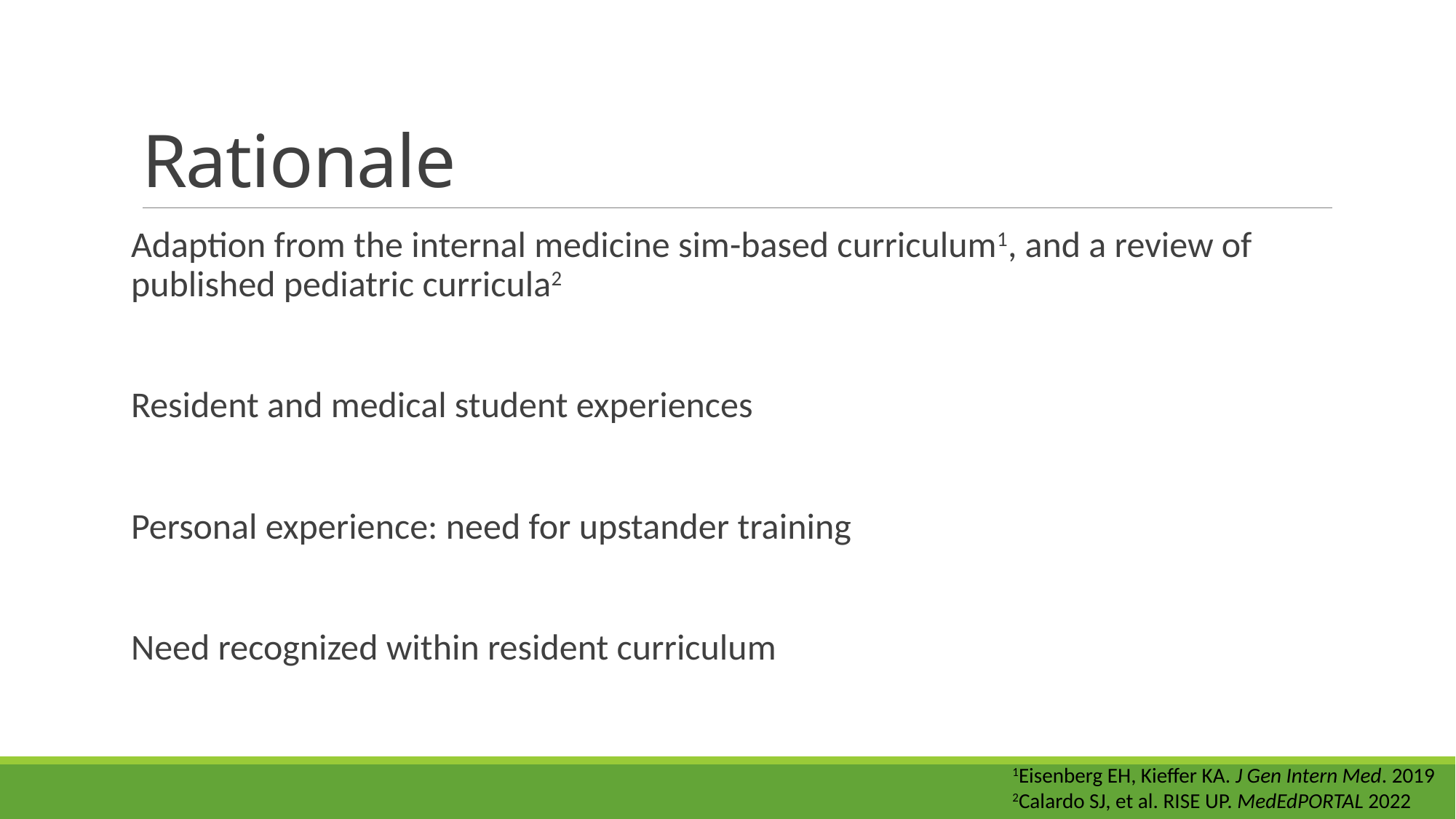

# Rationale
Adaption from the internal medicine sim-based curriculum1, and a review of published pediatric curricula2
Resident and medical student experiences
Personal experience: need for upstander training
Need recognized within resident curriculum
1Eisenberg EH, Kieffer KA. J Gen Intern Med. 2019
2Calardo SJ, et al. RISE UP. MedEdPORTAL 2022

## Slide 5
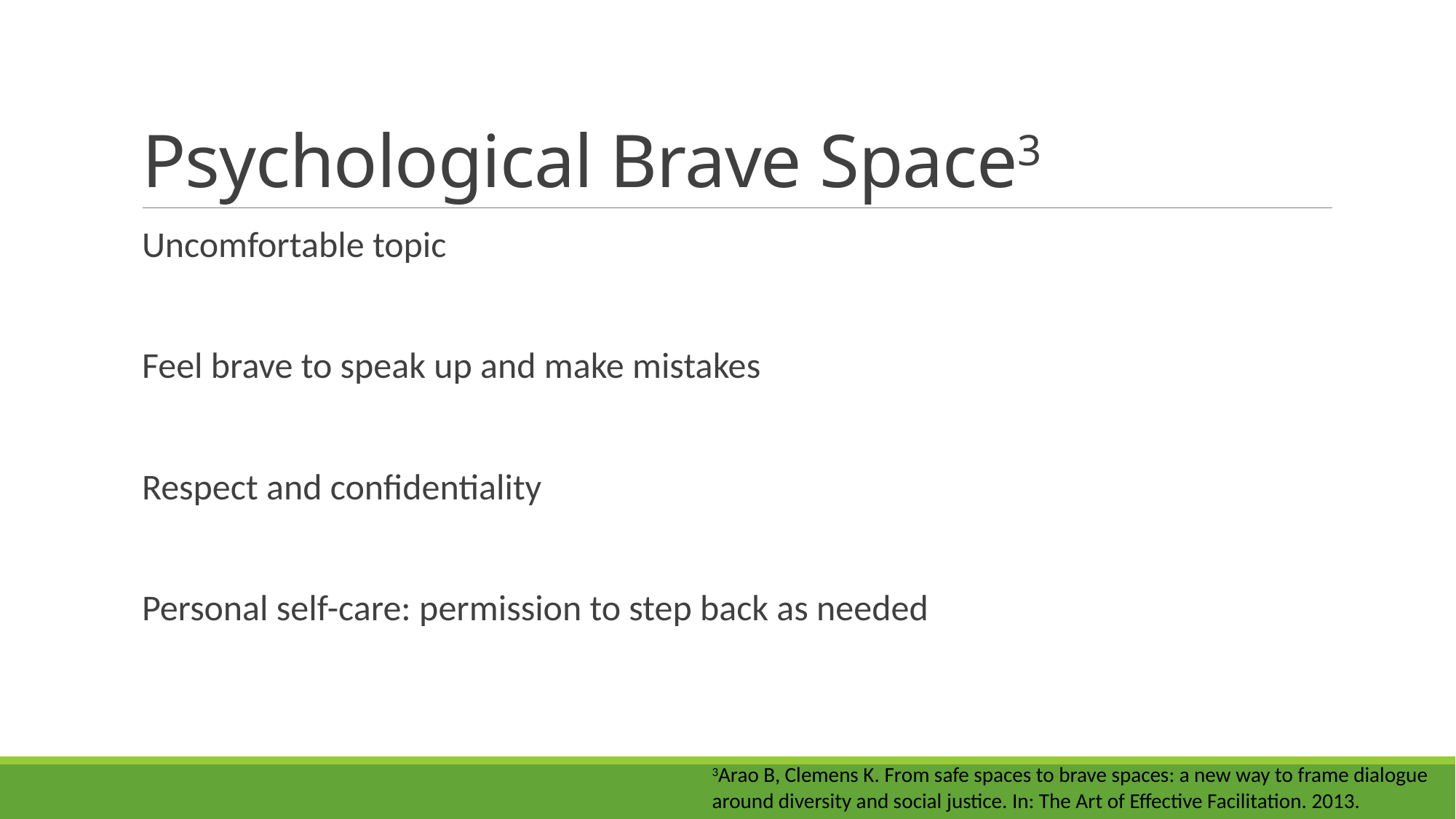

# Psychological Brave Space3
Uncomfortable topic
Feel brave to speak up and make mistakes
Respect and confidentiality
Personal self-care: permission to step back as needed
3Arao B, Clemens K. From safe spaces to brave spaces: a new way to frame dialogue around diversity and social justice. In: The Art of Effective Facilitation. 2013.

## Slide 6
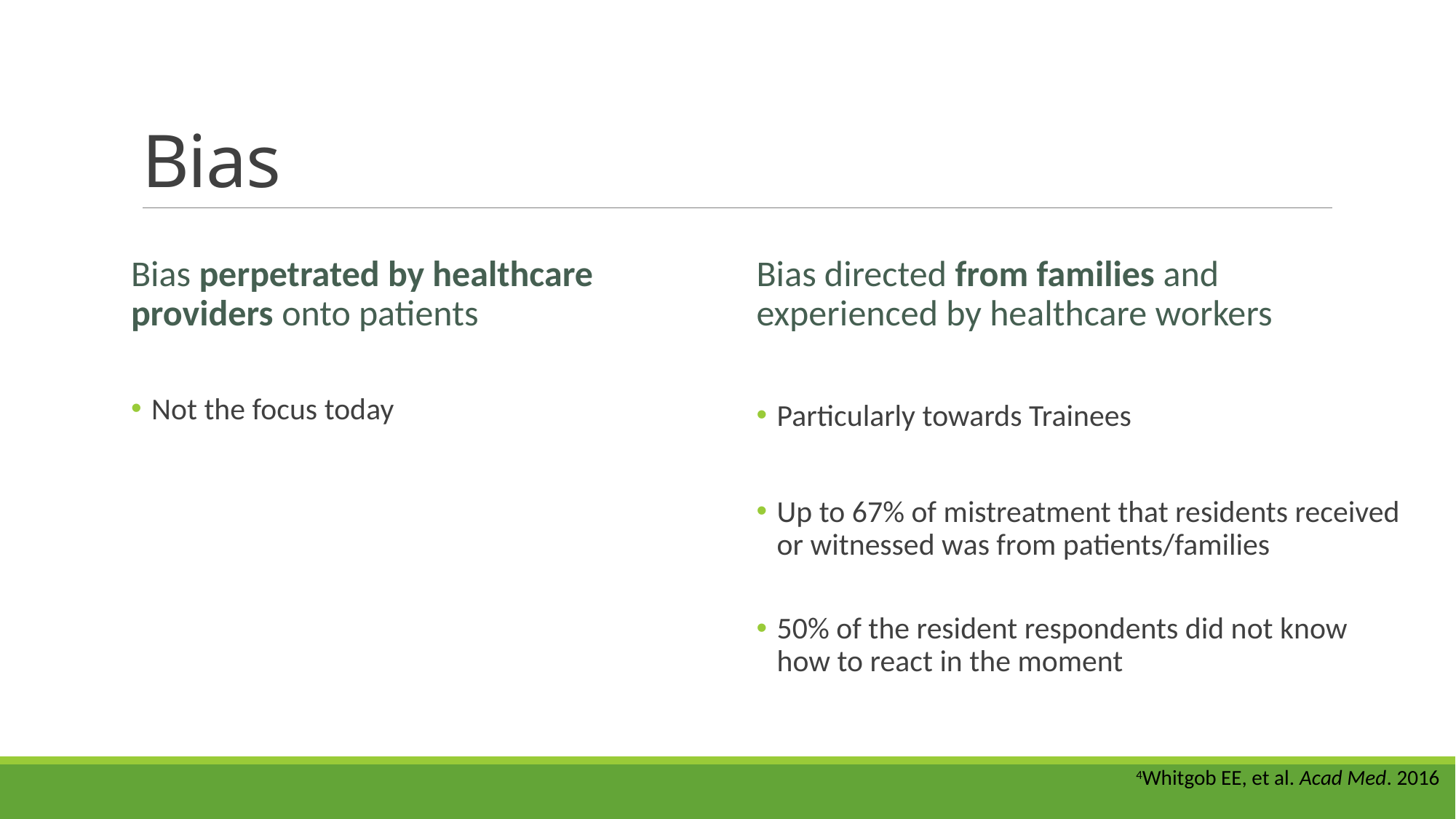

# Bias
Bias perpetrated by healthcare providers onto patients
Not the focus today
Bias directed from families and experienced by healthcare workers
Particularly towards Trainees
Up to 67% of mistreatment that residents received or witnessed was from patients/families
50% of the resident respondents did not know how to react in the moment
4Whitgob EE, et al. Acad Med. 2016

## Slide 7
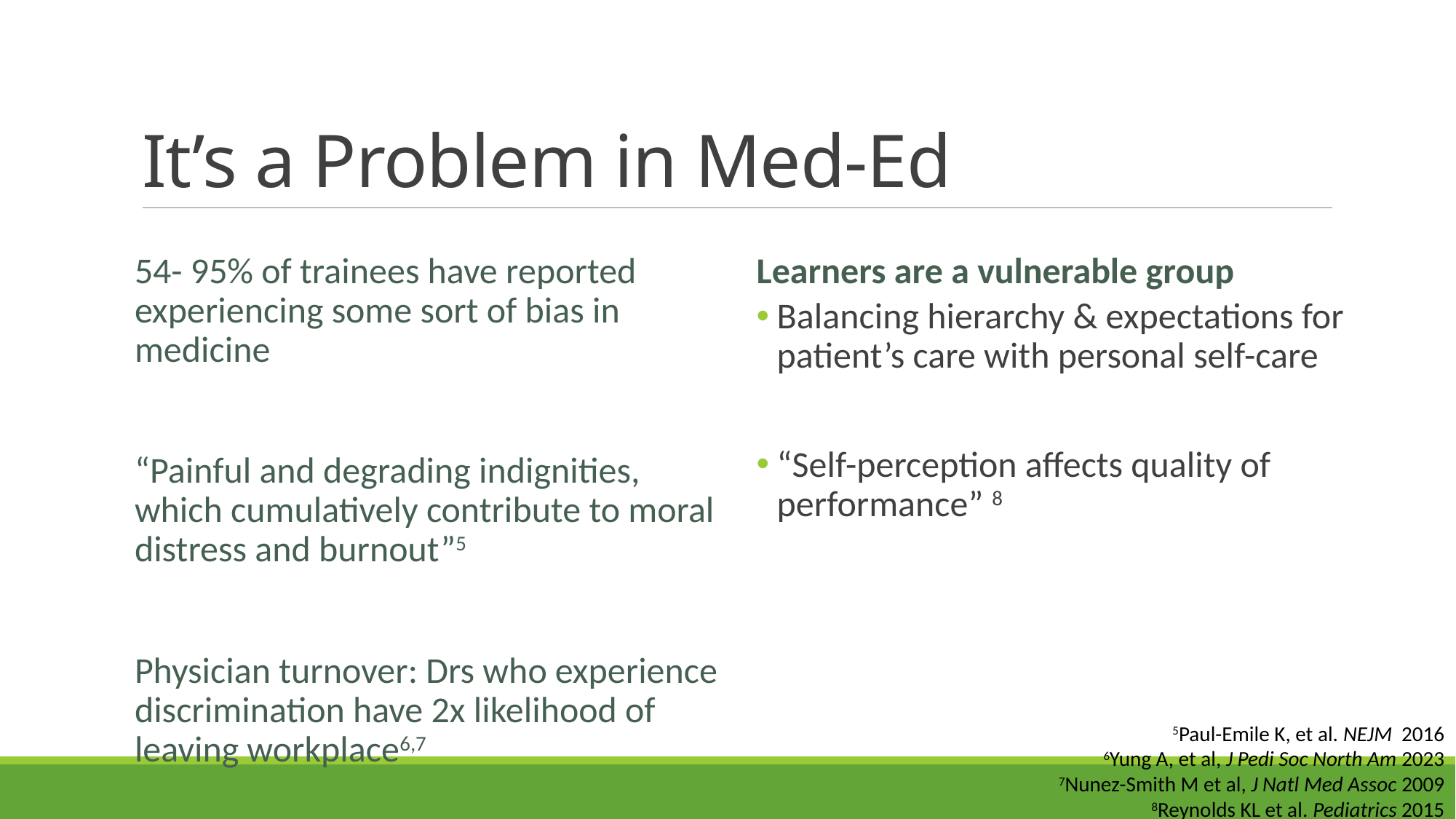

# It’s a Problem in Med-Ed
Learners are a vulnerable group
Balancing hierarchy & expectations for patient’s care with personal self-care
“Self-perception affects quality of performance” 8
54- 95% of trainees have reported experiencing some sort of bias in medicine
“Painful and degrading indignities, which cumulatively contribute to moral distress and burnout”5
Physician turnover: Drs who experience discrimination have 2x likelihood of leaving workplace6,7
5Paul-Emile K, et al. NEJM 2016
6Yung A, et al, J Pedi Soc North Am 2023
7Nunez-Smith M et al, J Natl Med Assoc 2009
8Reynolds KL et al. Pediatrics 2015

## Slide 8
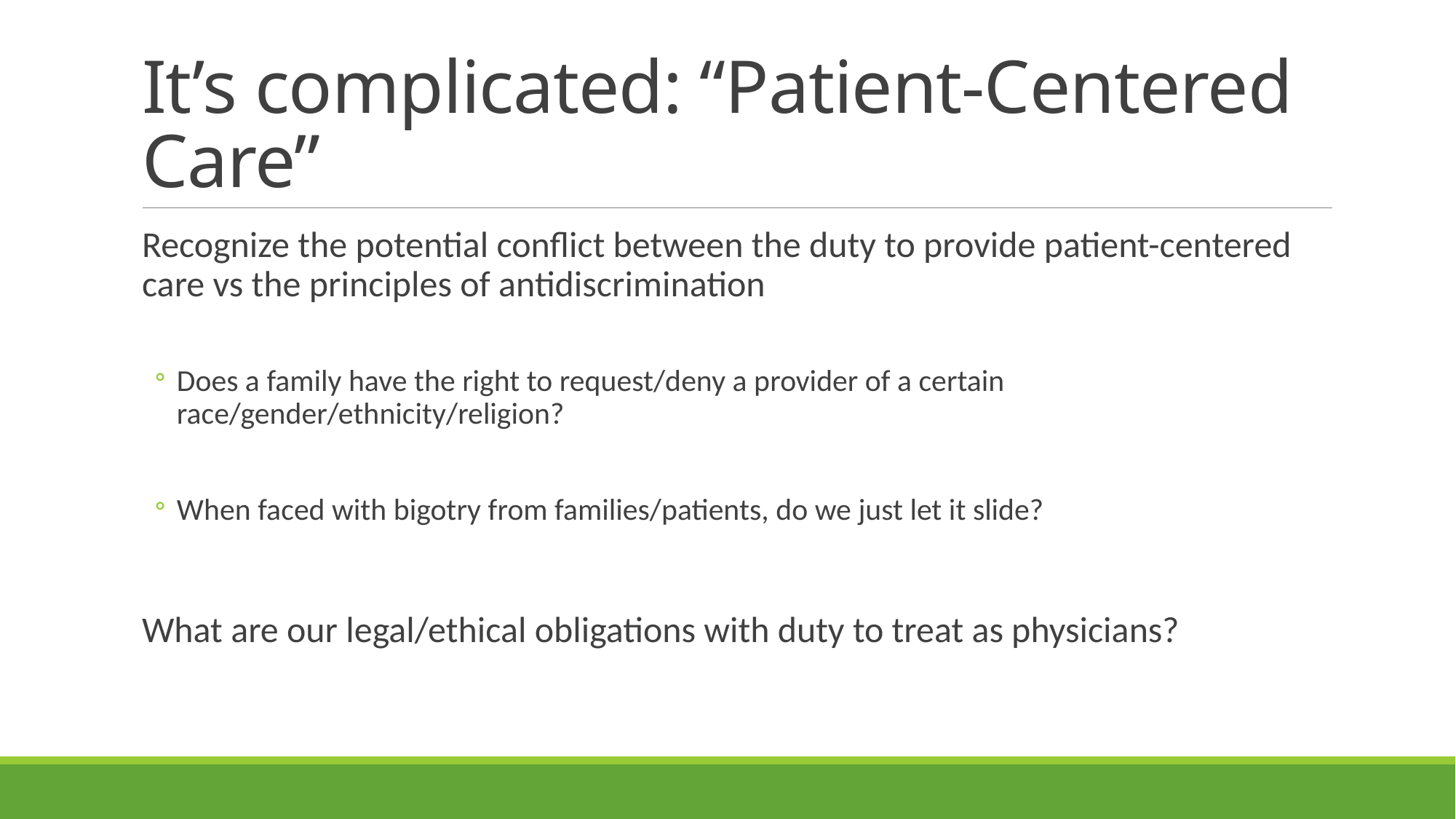

# It’s complicated: “Patient-Centered Care”
Recognize the potential conflict between the duty to provide patient-centered care vs the principles of antidiscrimination​
Does a family have the right to request/deny a provider of a certain race/gender/ethnicity/religion?
When faced with bigotry from families/patients, do we just let it slide?
What are our legal/ethical obligations with duty to treat as physicians?

## Slide 9
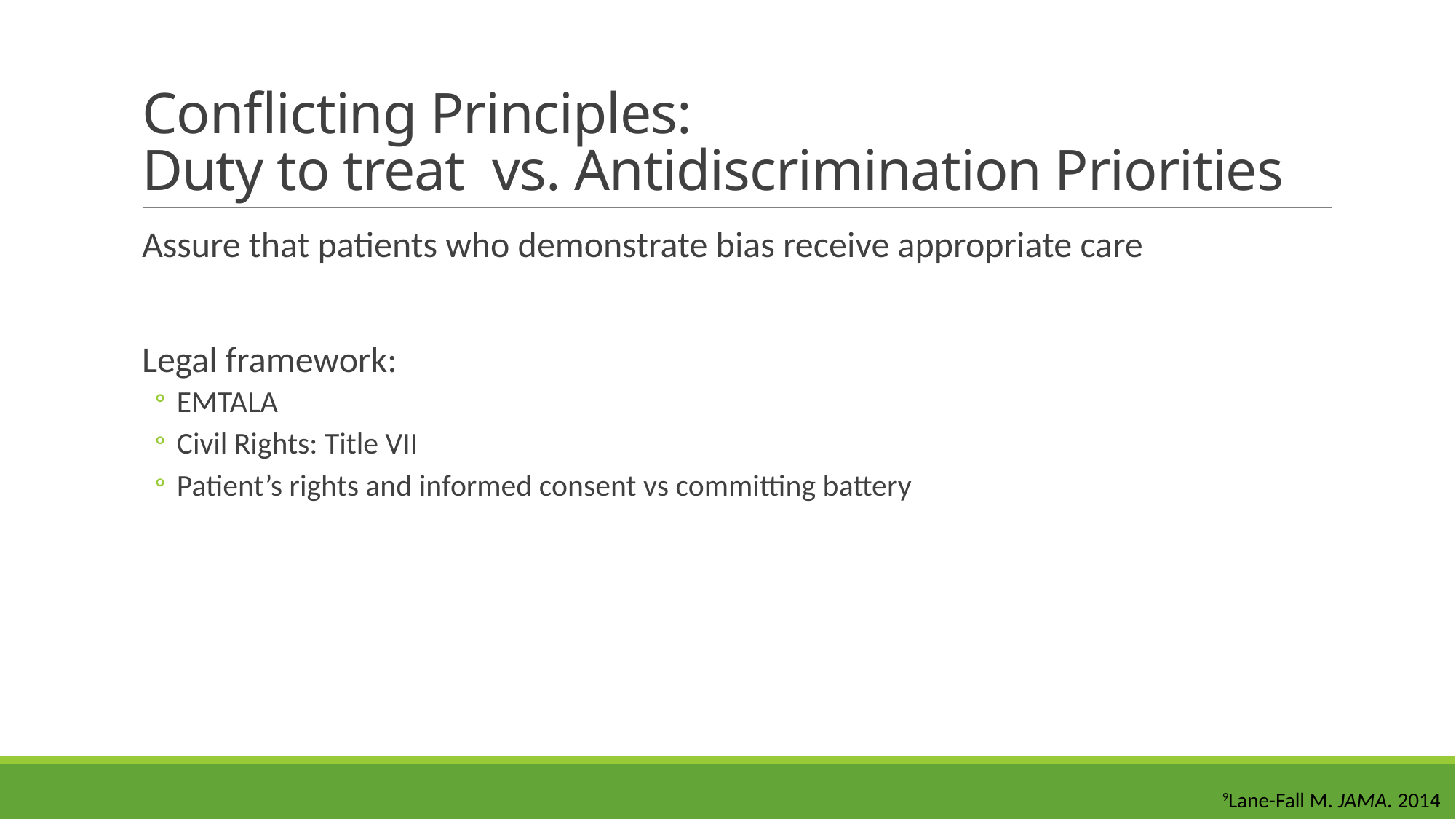

# Conflicting Principles: Duty to treat vs. Antidiscrimination Priorities
Assure that patients who demonstrate bias receive appropriate care
Legal framework:
EMTALA
Civil Rights: Title VII
​Patient’s rights and informed consent vs committing battery
9Lane-Fall M. JAMA. 2014

## Slide 10
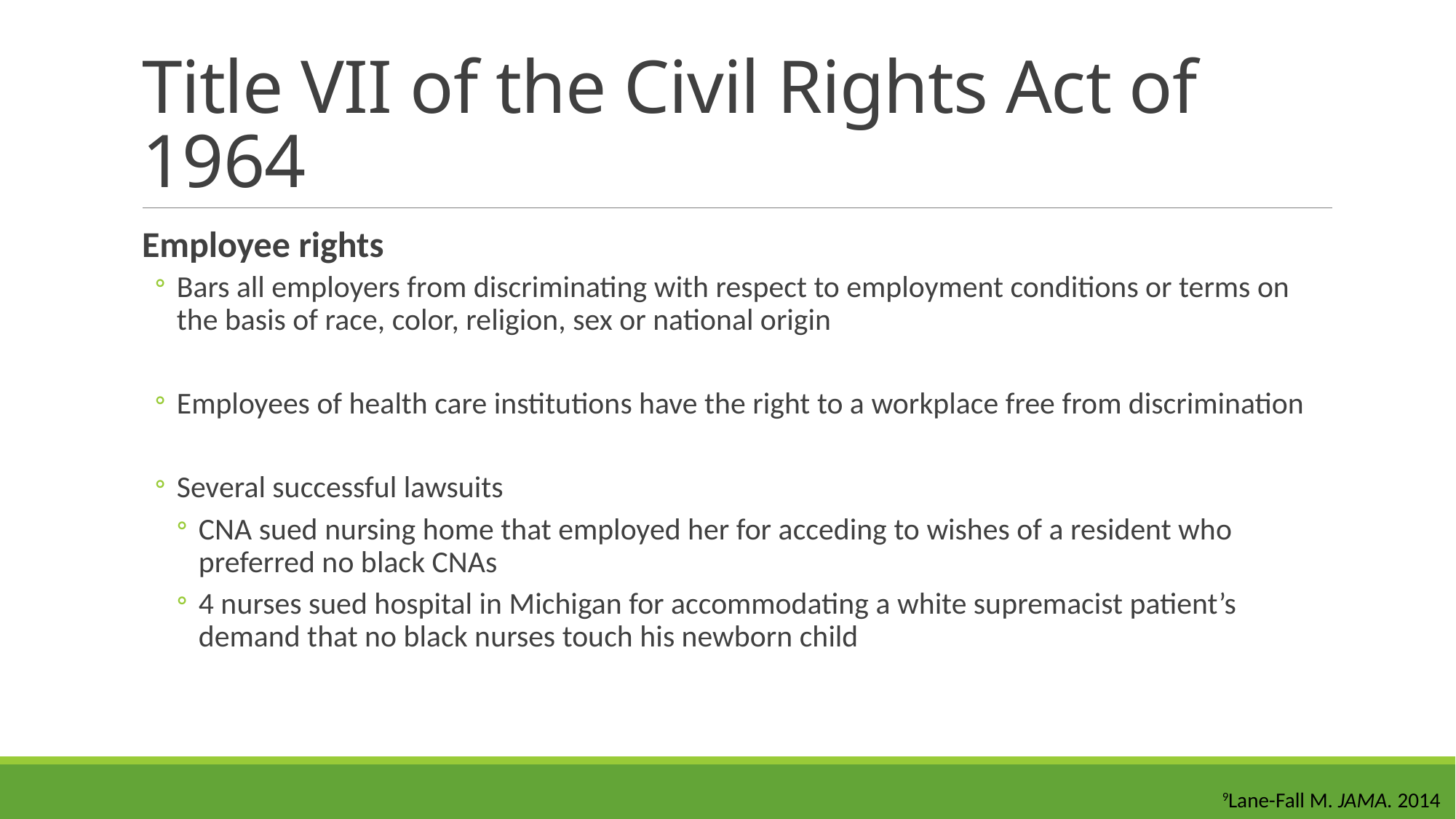

# Title VII of the Civil Rights Act of 1964
Employee rights
Bars all employers from discriminating with respect to employment conditions or terms on the basis of race, color, religion, sex or national origin
Employees of health care institutions have the right to a workplace free from discrimination
Several successful lawsuits
CNA sued nursing home that employed her for acceding to wishes of a resident who preferred no black CNAs
4 nurses sued hospital in Michigan for accommodating a white supremacist patient’s demand that no black nurses touch his newborn child
9Lane-Fall M. JAMA. 2014

## Slide 11
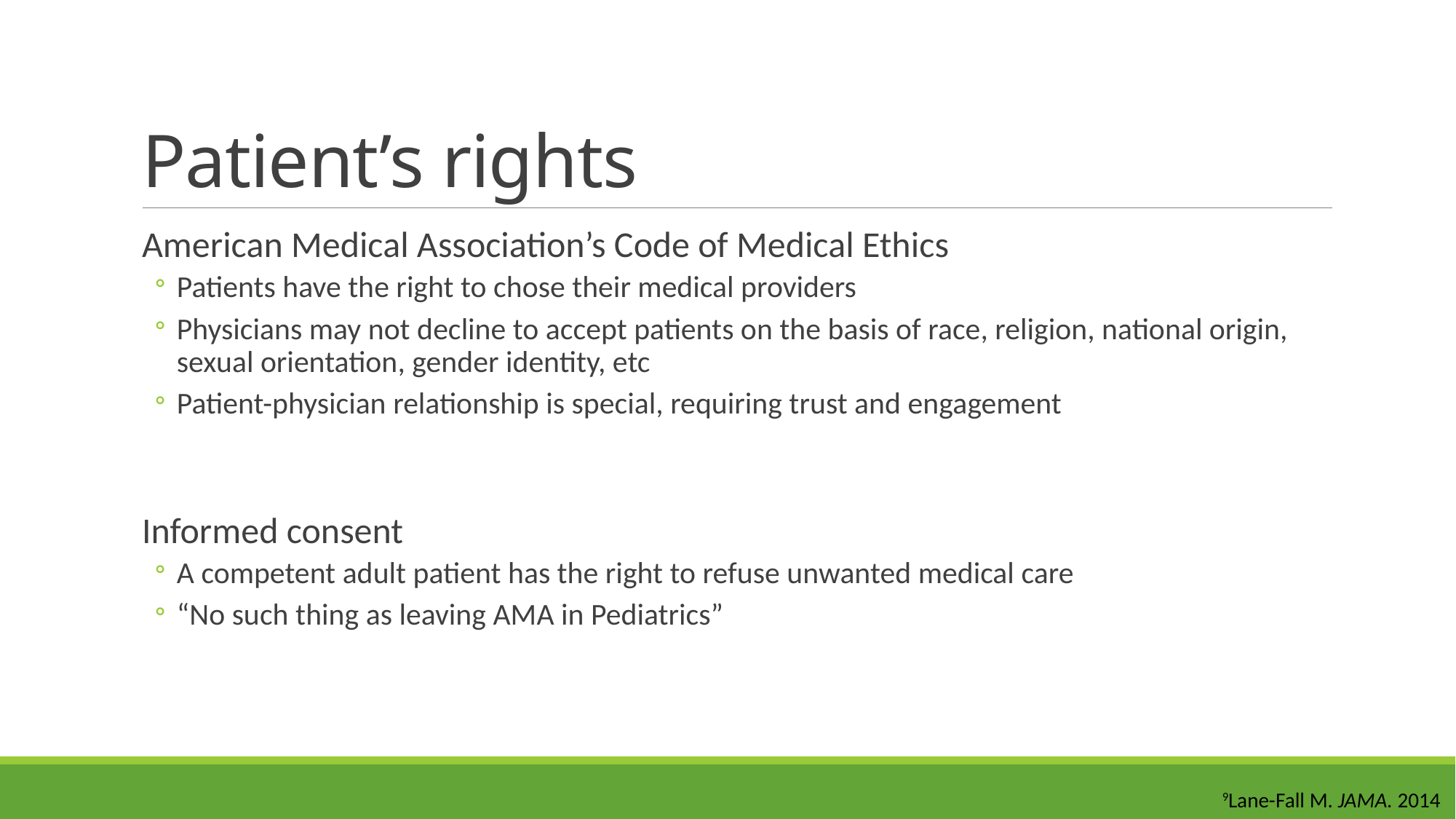

# Patient’s rights
American Medical Association’s Code of Medical Ethics
Patients have the right to chose their medical providers
Physicians may not decline to accept patients on the basis of race, religion, national origin, sexual orientation, gender identity, etc
Patient-physician relationship is special, requiring trust and engagement
Informed consent
A competent adult patient has the right to refuse unwanted medical care
“No such thing as leaving AMA in Pediatrics”
9Lane-Fall M. JAMA. 2014

## Slide 12
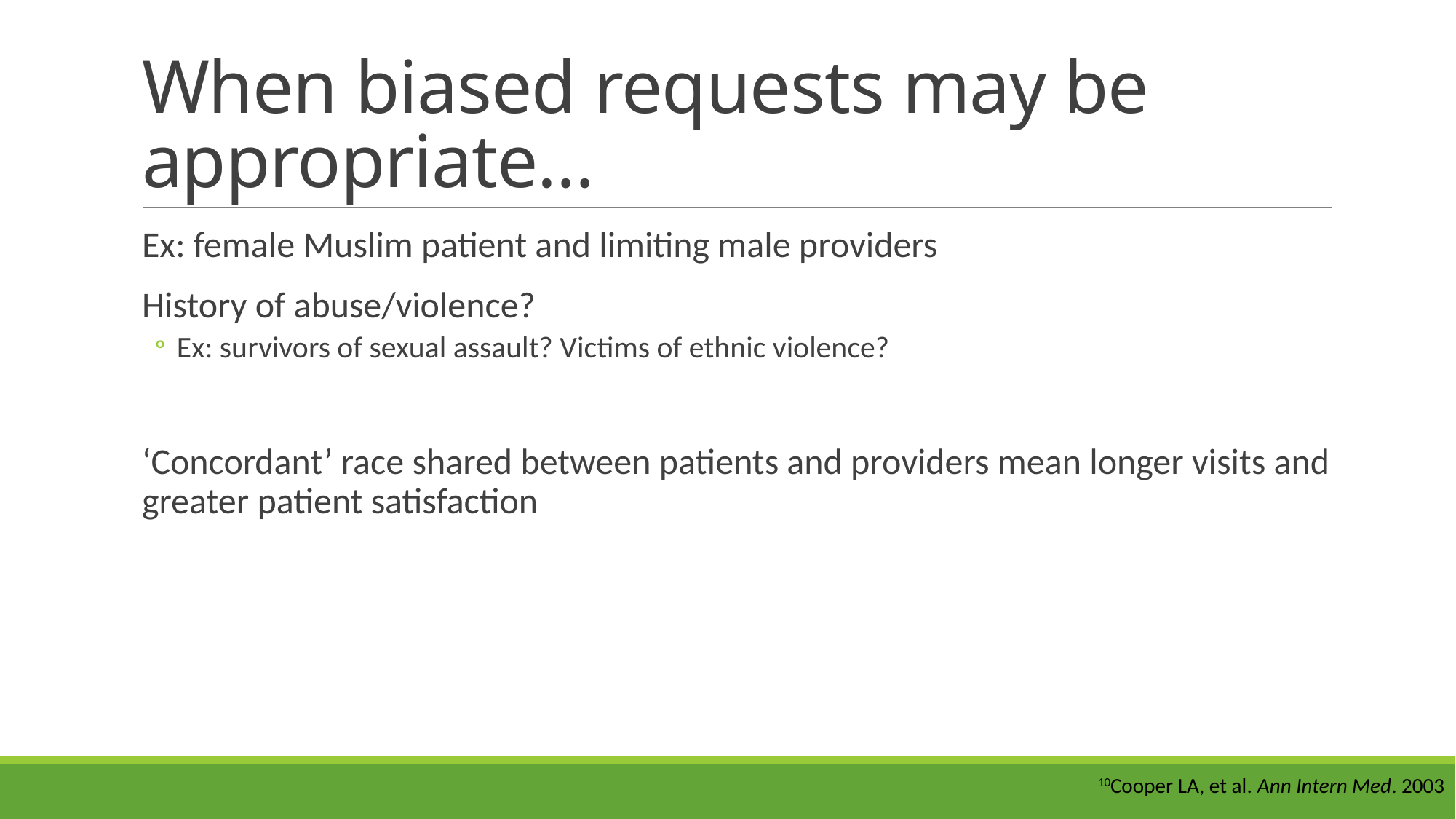

# When biased requests may be appropriate…
Ex: female Muslim patient and limiting male providers
History of abuse/violence?
Ex: survivors of sexual assault? Victims of ethnic violence?
‘Concordant’ race shared between patients and providers mean longer visits and greater patient satisfaction
10Cooper LA, et al. Ann Intern Med. 2003

## Slide 13
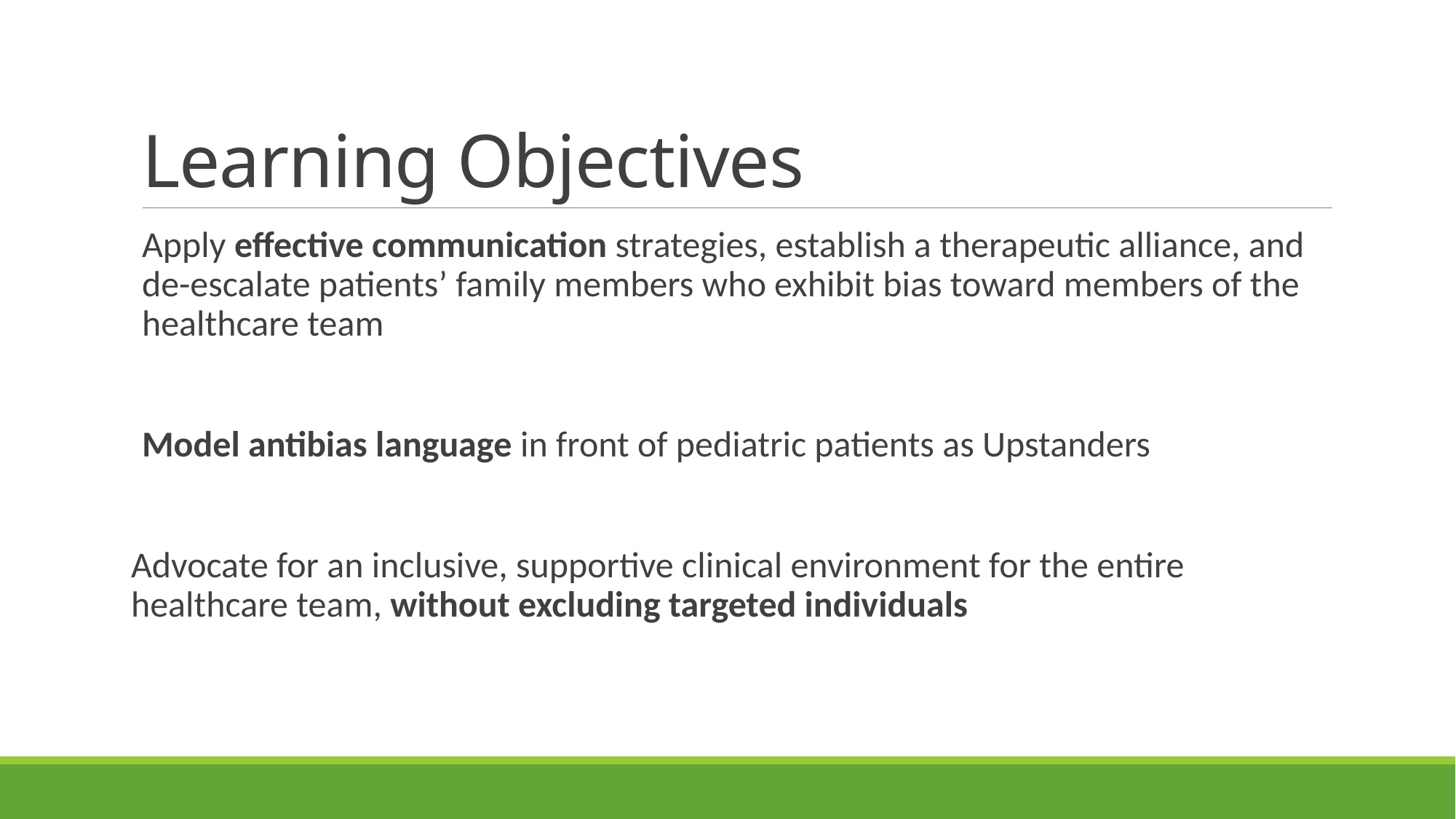

# Learning Objectives
Apply effective communication strategies, establish a therapeutic alliance, and de-escalate patients’ family members who exhibit bias toward members of the healthcare team ​
Model antibias language in front of pediatric patients​ as Upstanders
Advocate for an inclusive, supportive clinical environment for the entire healthcare team, without excluding targeted individuals

## Slide 14
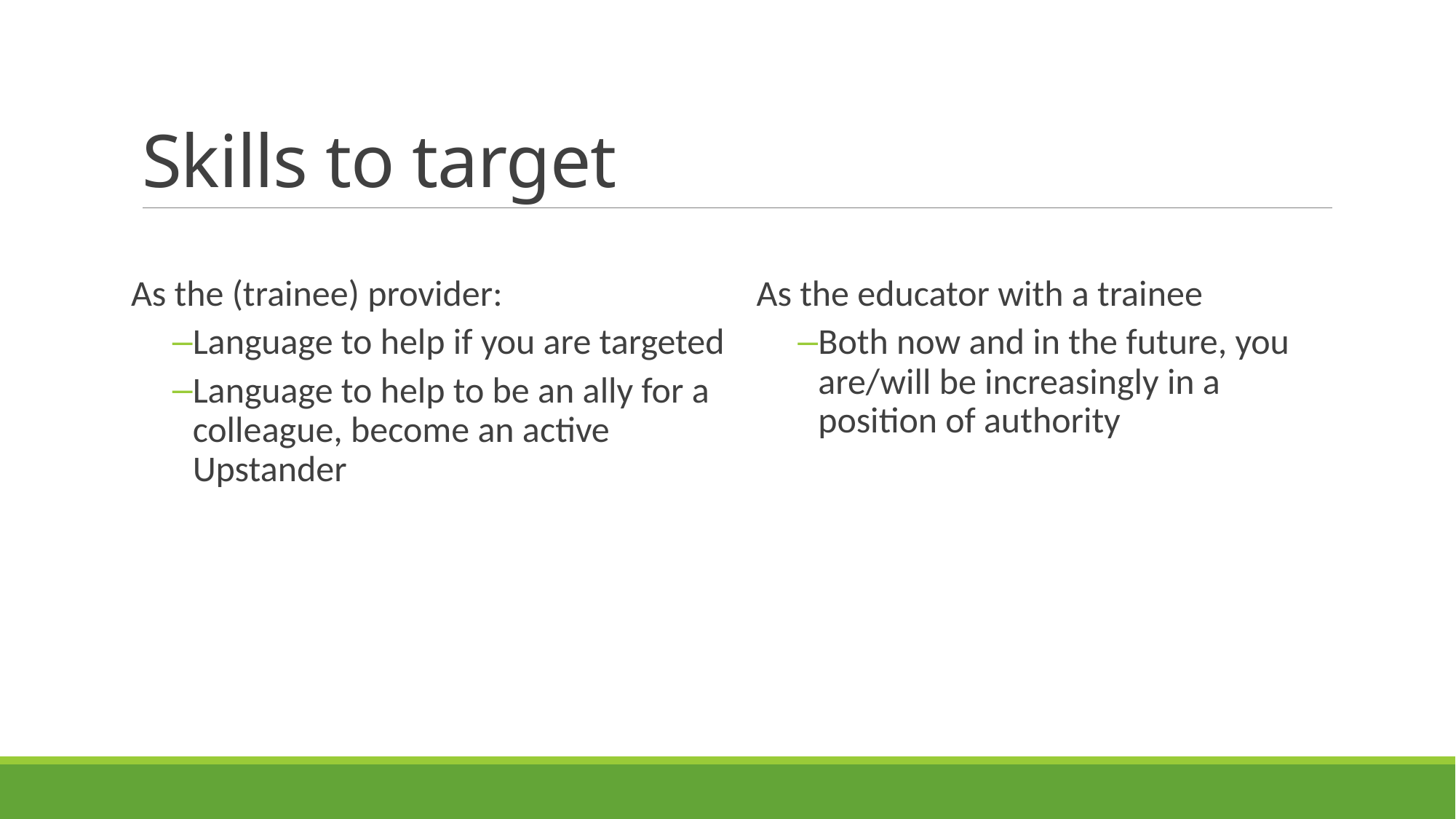

# Skills to target
As the (trainee) provider:
Language to help if you are targeted
Language to help to be an ally for a colleague, become an active Upstander
As the educator with a trainee
Both now and in the future, you are/will be increasingly in a position of authority

## Slide 15
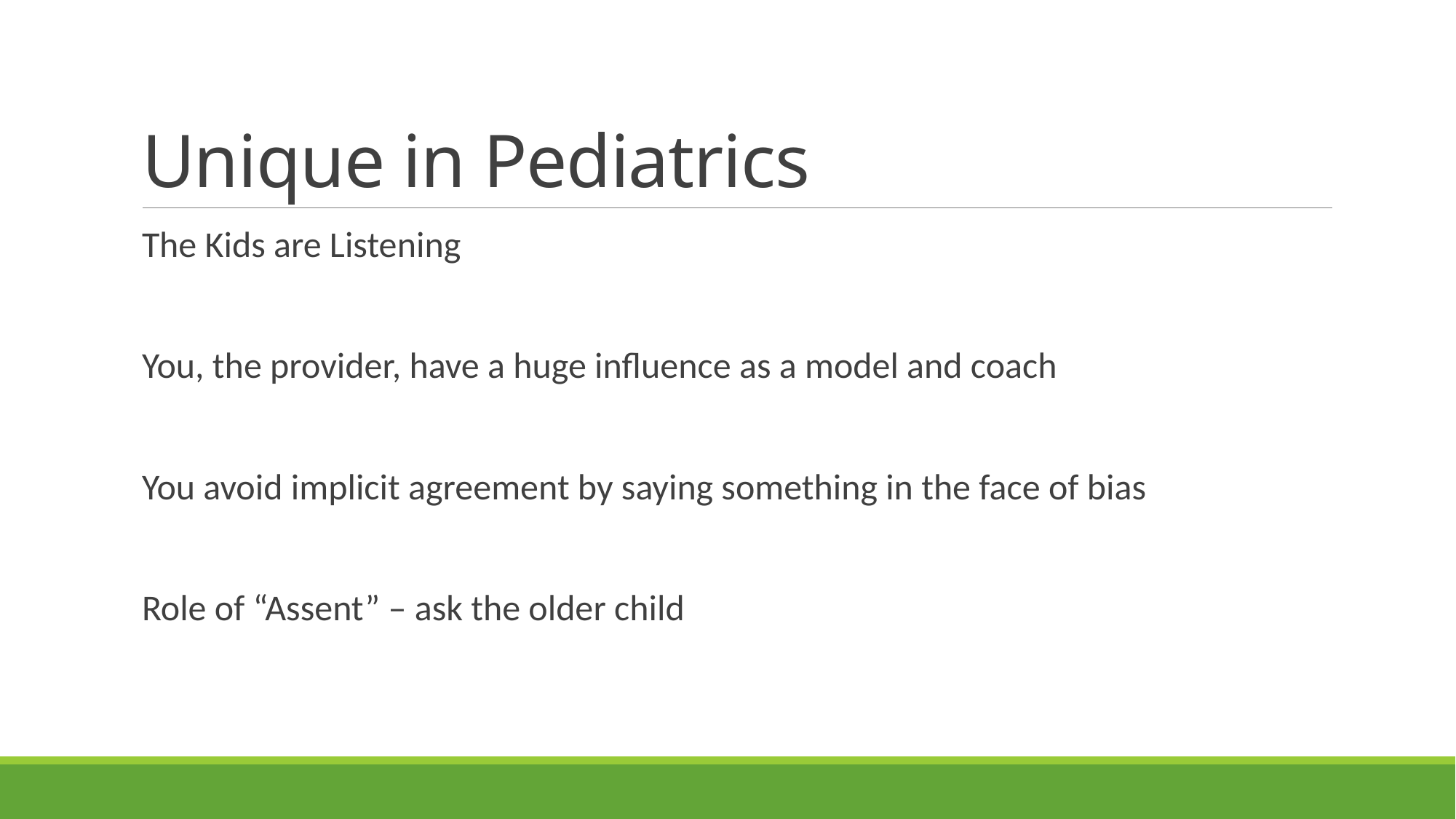

# Unique in Pediatrics
The Kids are Listening
You, the provider, have a huge influence as a model and coach
You avoid implicit agreement by saying something in the face of bias
Role of “Assent” – ask the older child

## Slide 16
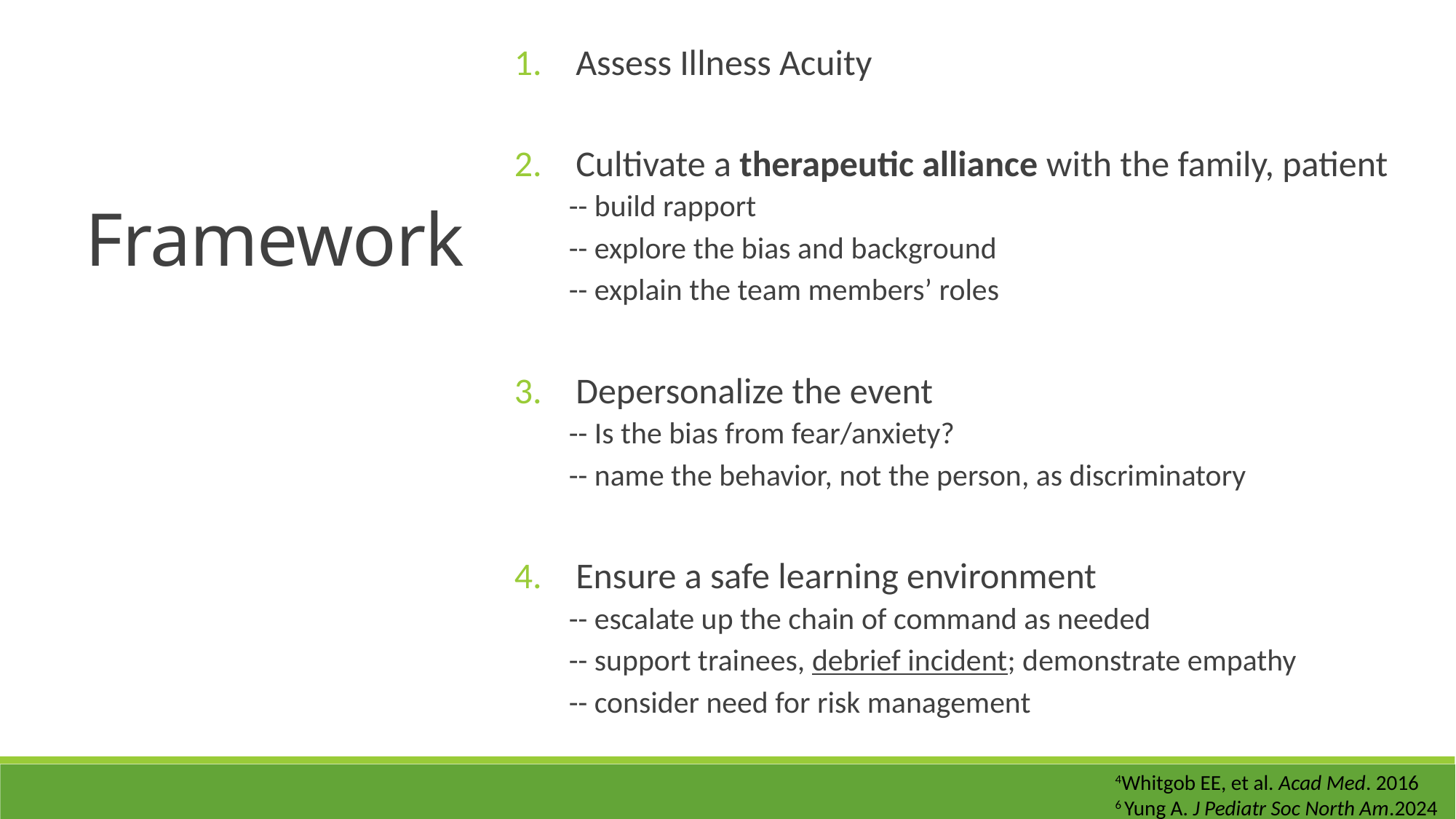

Assess Illness Acuity
Cultivate a therapeutic alliance with the family, patient
-- build rapport
-- explore the bias and background
-- explain the team members’ roles
Depersonalize the event
-- Is the bias from fear/anxiety?
-- name the behavior, not the person, as discriminatory
Ensure a safe learning environment
-- escalate up the chain of command as needed
-- support trainees, debrief incident; demonstrate empathy
-- consider need for risk management
Framework
4Whitgob EE, et al. Acad Med. 2016
6 Yung A. J Pediatr Soc North Am.2024

## Slide 17
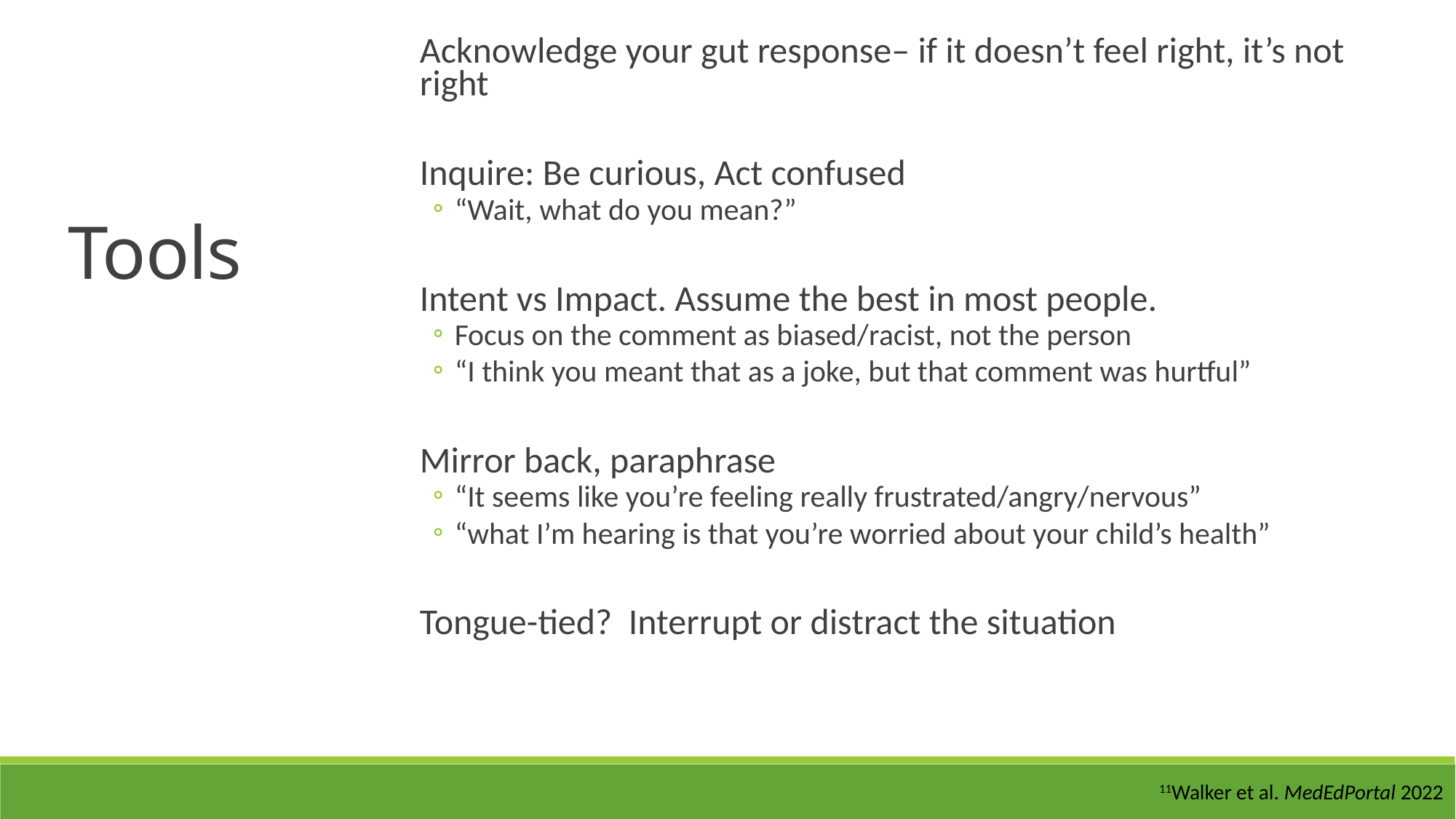

Acknowledge your gut response– if it doesn’t feel right, it’s not right
Inquire: Be curious, Act confused
“Wait, what do you mean?”
Intent vs Impact. Assume the best in most people.
Focus on the comment as biased/racist, not the person
“I think you meant that as a joke, but that comment was hurtful”
Mirror back, paraphrase
“It seems like you’re feeling really frustrated/angry/nervous”
“what I’m hearing is that you’re worried about your child’s health”
Tongue-tied? Interrupt or distract the situation
Tools
11Walker et al. MedEdPortal 2022

## Slide 18
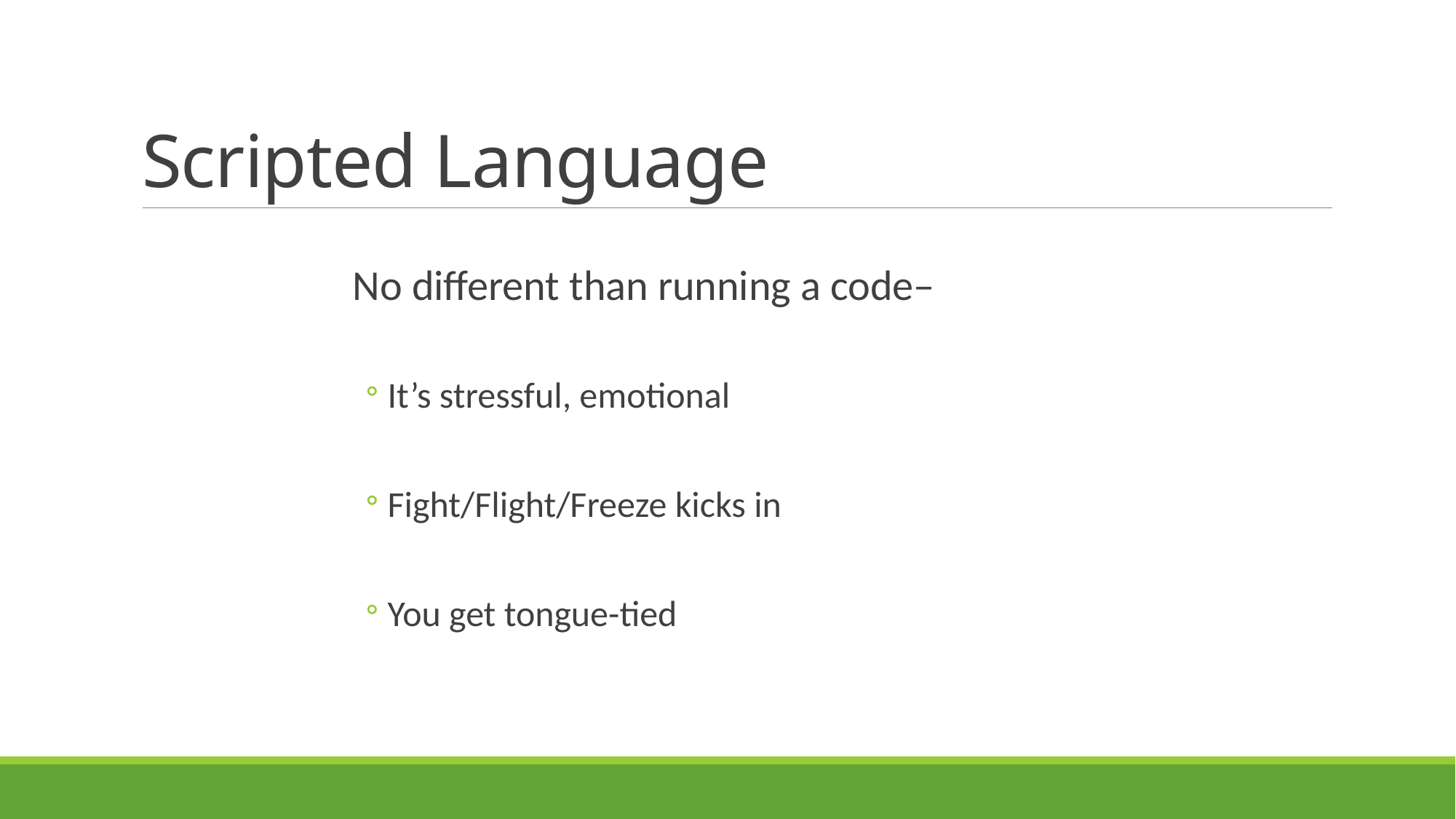

# Scripted Language
No different than running a code–
It’s stressful, emotional
Fight/Flight/Freeze kicks in
You get tongue-tied

## Slide 19
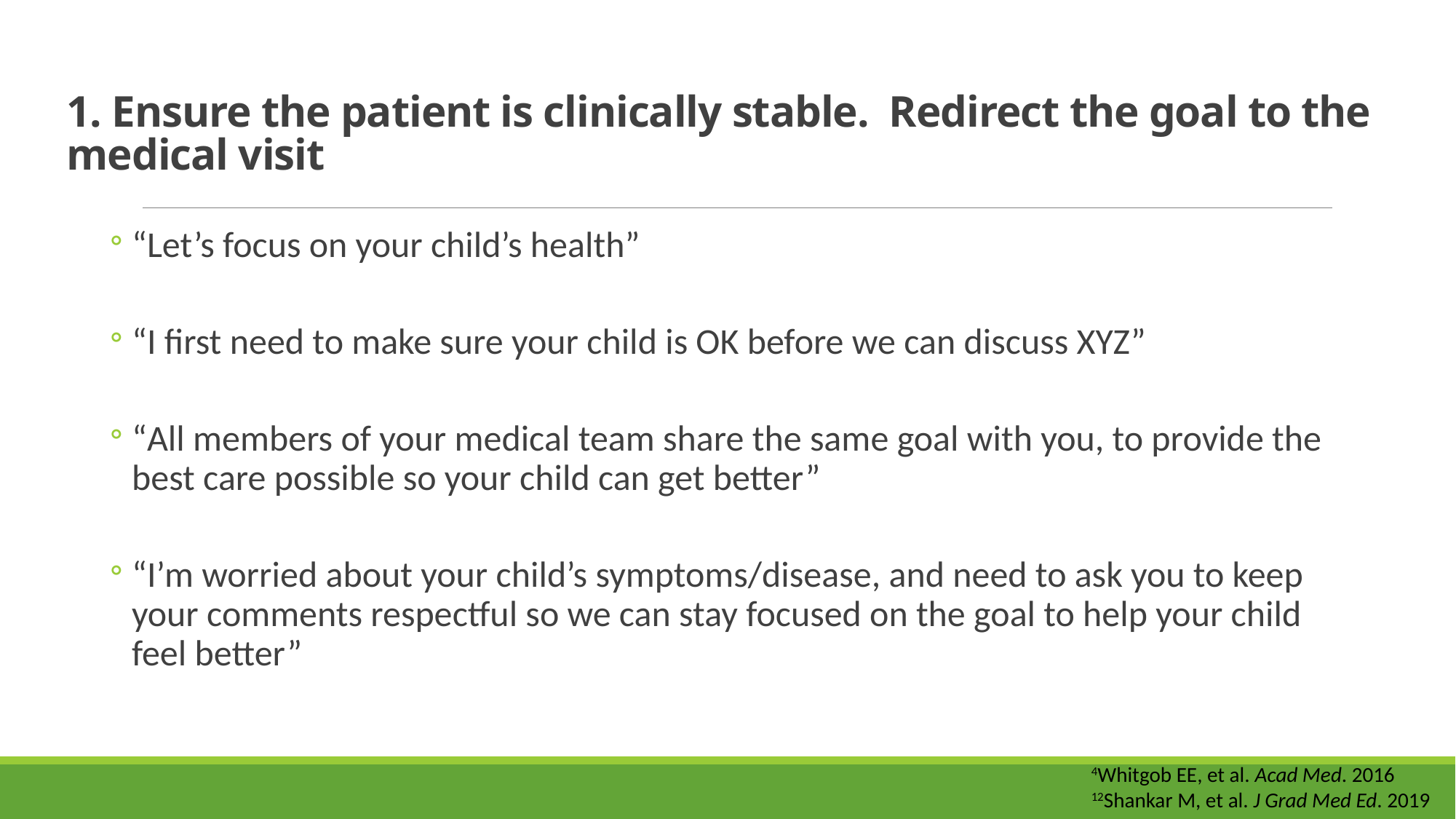

# 1. Ensure the patient is clinically stable. Redirect the goal to the medical visit
“Let’s focus on your child’s health”
“I first need to make sure your child is OK before we can discuss XYZ”
“All members of your medical team share the same goal with you, to provide the best care possible so your child can get better”
“I’m worried about your child’s symptoms/disease, and need to ask you to keep your comments respectful so we can stay focused on the goal to help your child feel better”
4Whitgob EE, et al. Acad Med. 2016
12Shankar M, et al. J Grad Med Ed. 2019

## Slide 20
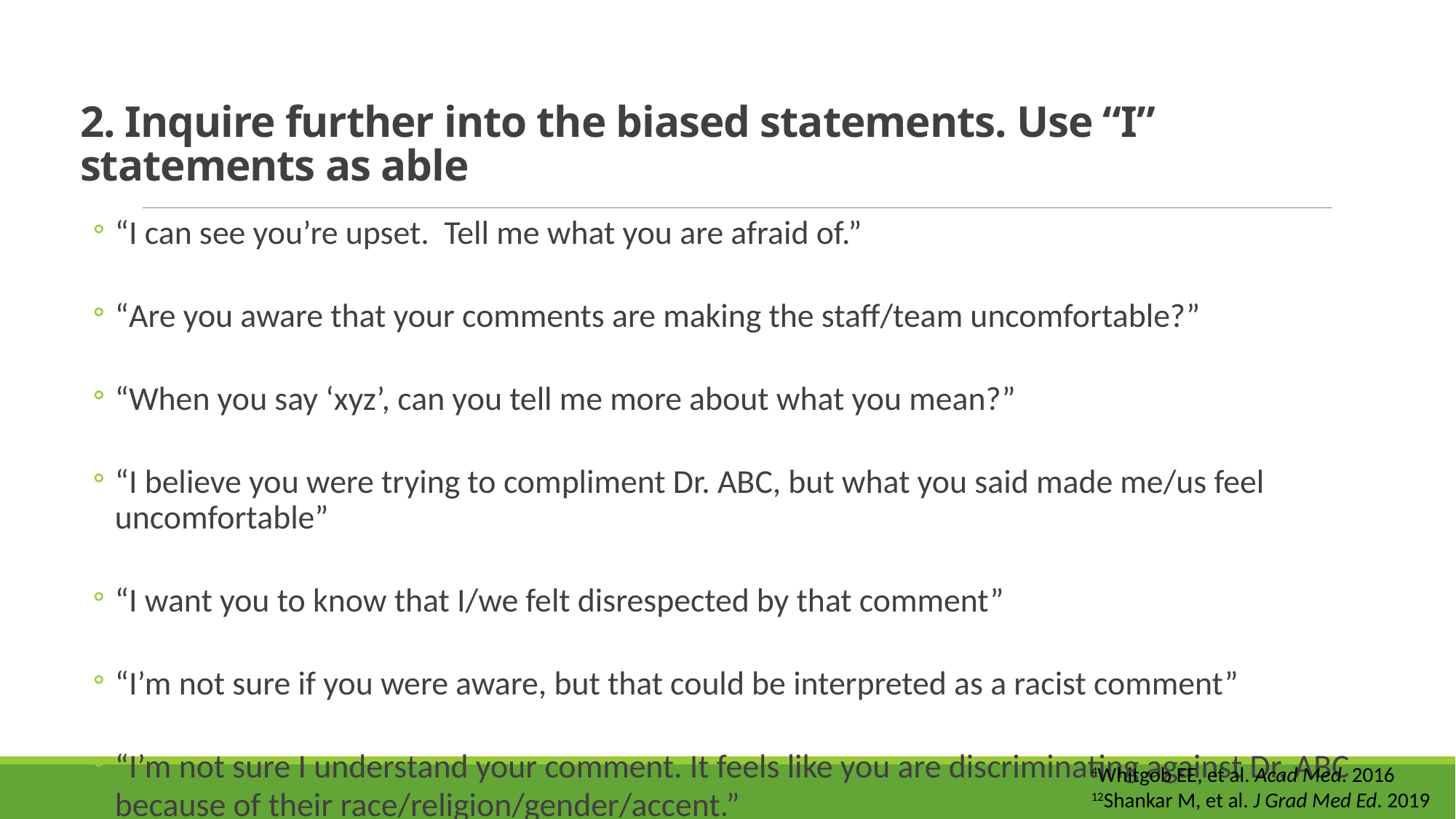

# 2. Inquire further into the biased statements. Use “I” statements as able
“I can see you’re upset. Tell me what you are afraid of.”
“Are you aware that your comments are making the staff/team uncomfortable?”
“When you say ‘xyz’, can you tell me more about what you mean?”
“I believe you were trying to compliment Dr. ABC, but what you said made me/us feel uncomfortable”
“I want you to know that I/we felt disrespected by that comment”
“I’m not sure if you were aware, but that could be interpreted as a racist comment”
“I’m not sure I understand your comment. It feels like you are discriminating against Dr. ABC because of their race/religion/gender/accent.”
4Whitgob EE, et al. Acad Med. 2016
12Shankar M, et al. J Grad Med Ed. 2019

## Slide 21
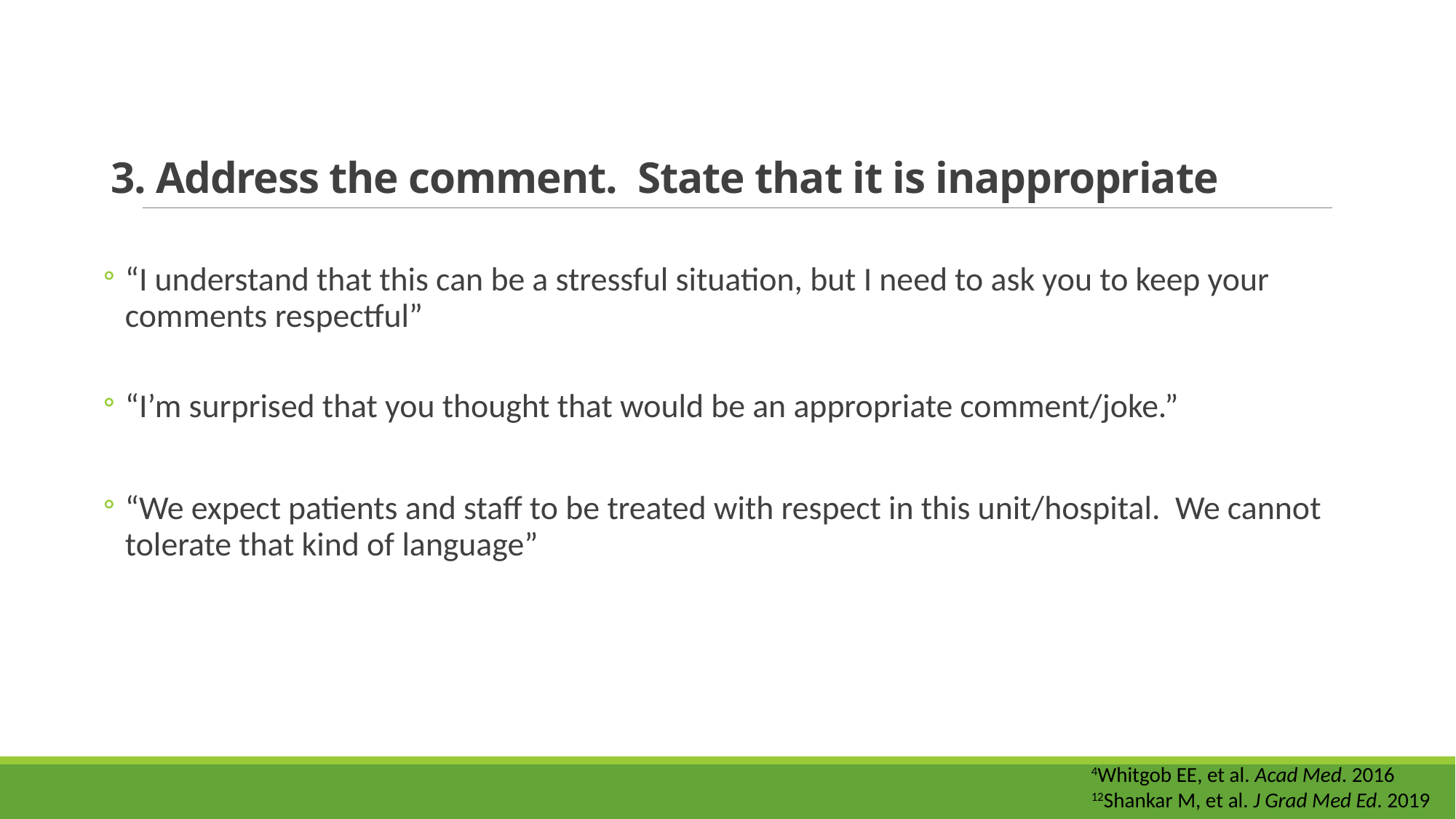

# 3. Address the comment. State that it is inappropriate
“I understand that this can be a stressful situation, but I need to ask you to keep your comments respectful”
“I’m surprised that you thought that would be an appropriate comment/joke.”
“We expect patients and staff to be treated with respect in this unit/hospital. We cannot tolerate that kind of language”
4Whitgob EE, et al. Acad Med. 2016
12Shankar M, et al. J Grad Med Ed. 2019

## Slide 22
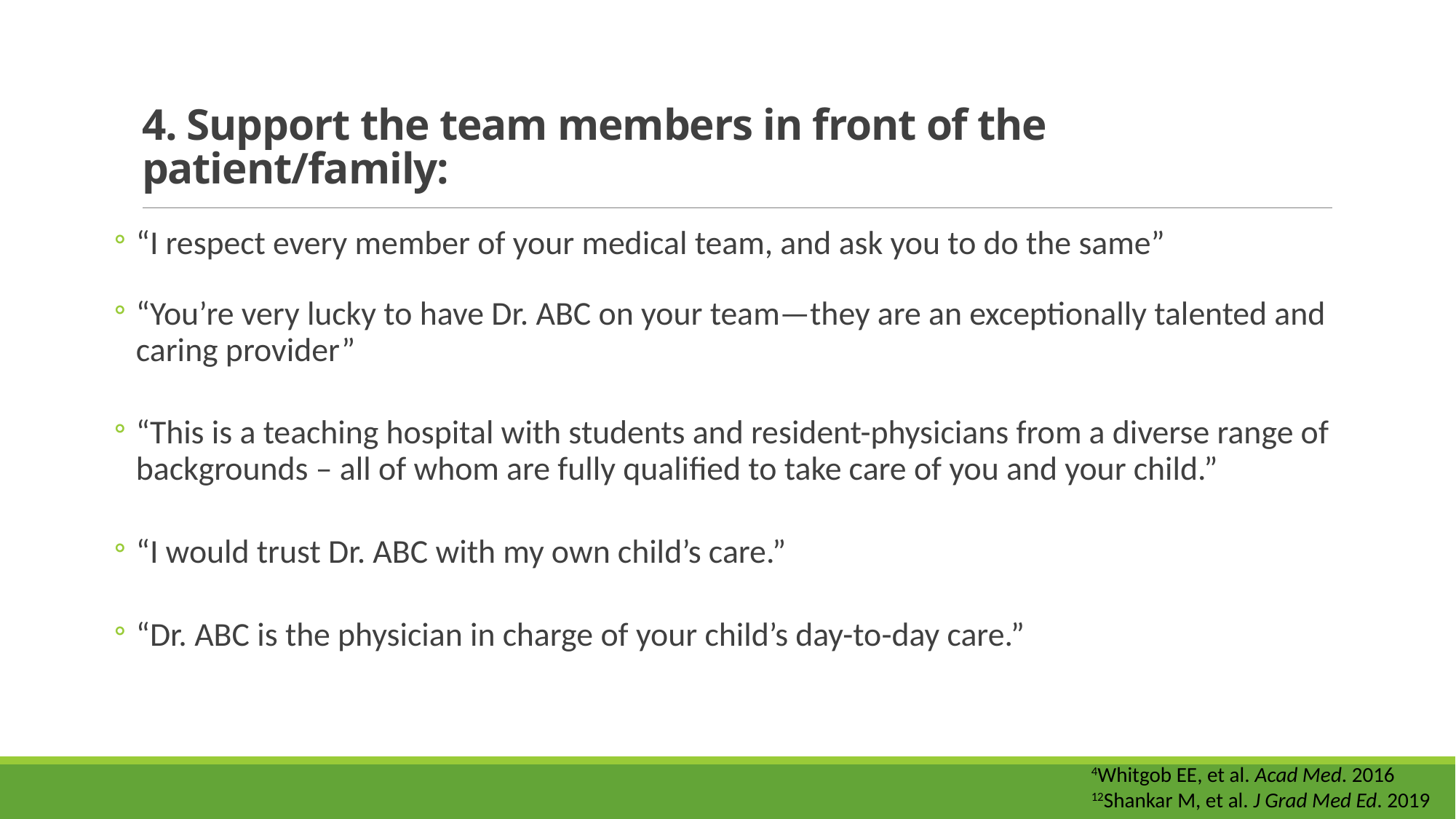

# 4. Support the team members in front of the patient/family:
“I respect every member of your medical team, and ask you to do the same”
“You’re very lucky to have Dr. ABC on your team—they are an exceptionally talented and caring provider”
“This is a teaching hospital with students and resident-physicians from a diverse range of backgrounds – all of whom are fully qualified to take care of you and your child.”
“I would trust Dr. ABC with my own child’s care.”
“Dr. ABC is the physician in charge of your child’s day-to-day care.”
4Whitgob EE, et al. Acad Med. 2016
12Shankar M, et al. J Grad Med Ed. 2019

## Slide 23
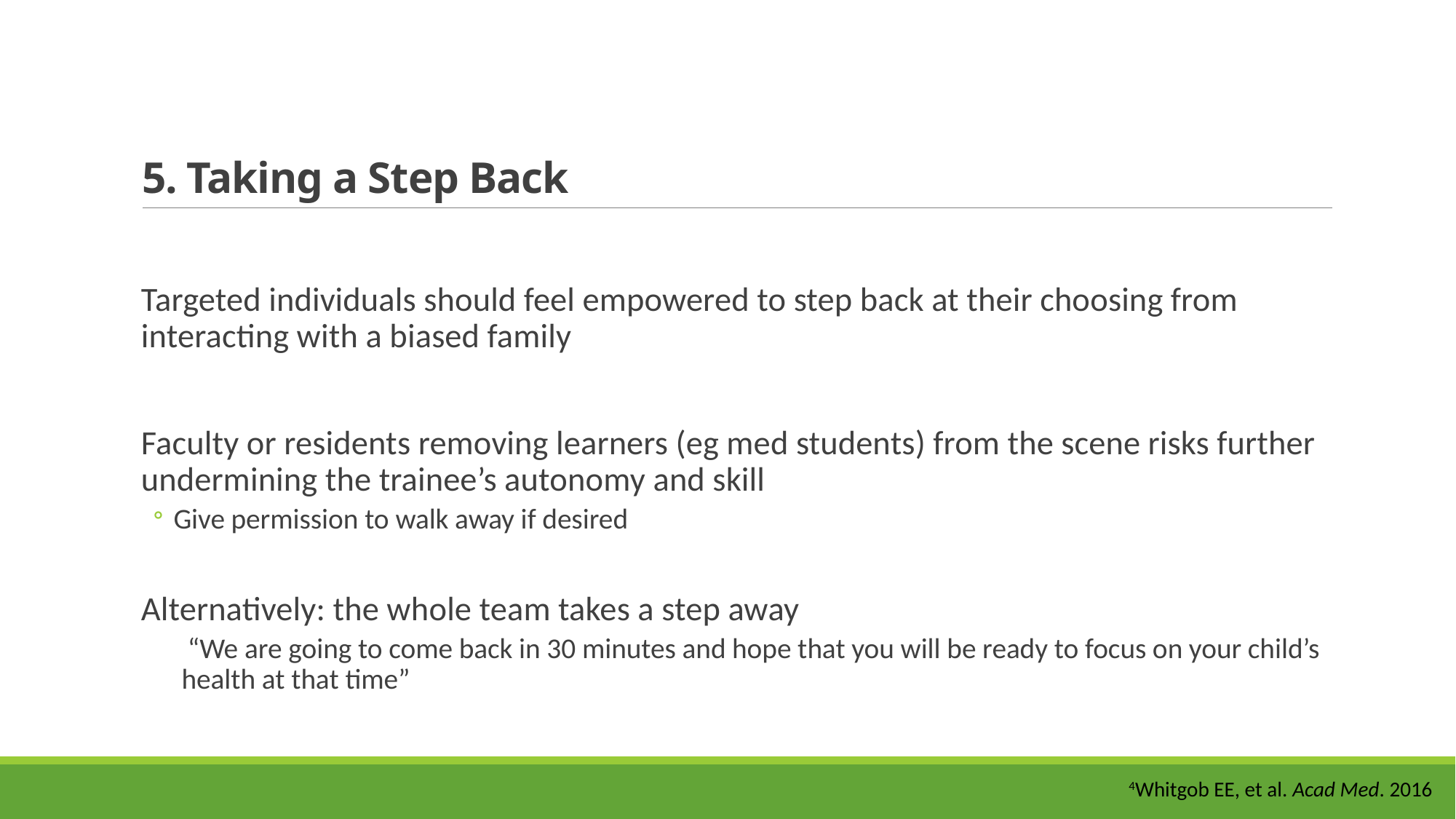

# 5. Taking a Step Back
Targeted individuals should feel empowered to step back at their choosing from interacting with a biased family
Faculty or residents removing learners (eg med students) from the scene risks further undermining the trainee’s autonomy and skill
Give permission to walk away if desired
Alternatively: the whole team takes a step away
 “We are going to come back in 30 minutes and hope that you will be ready to focus on your child’s health at that time”
4Whitgob EE, et al. Acad Med. 2016

## Slide 24
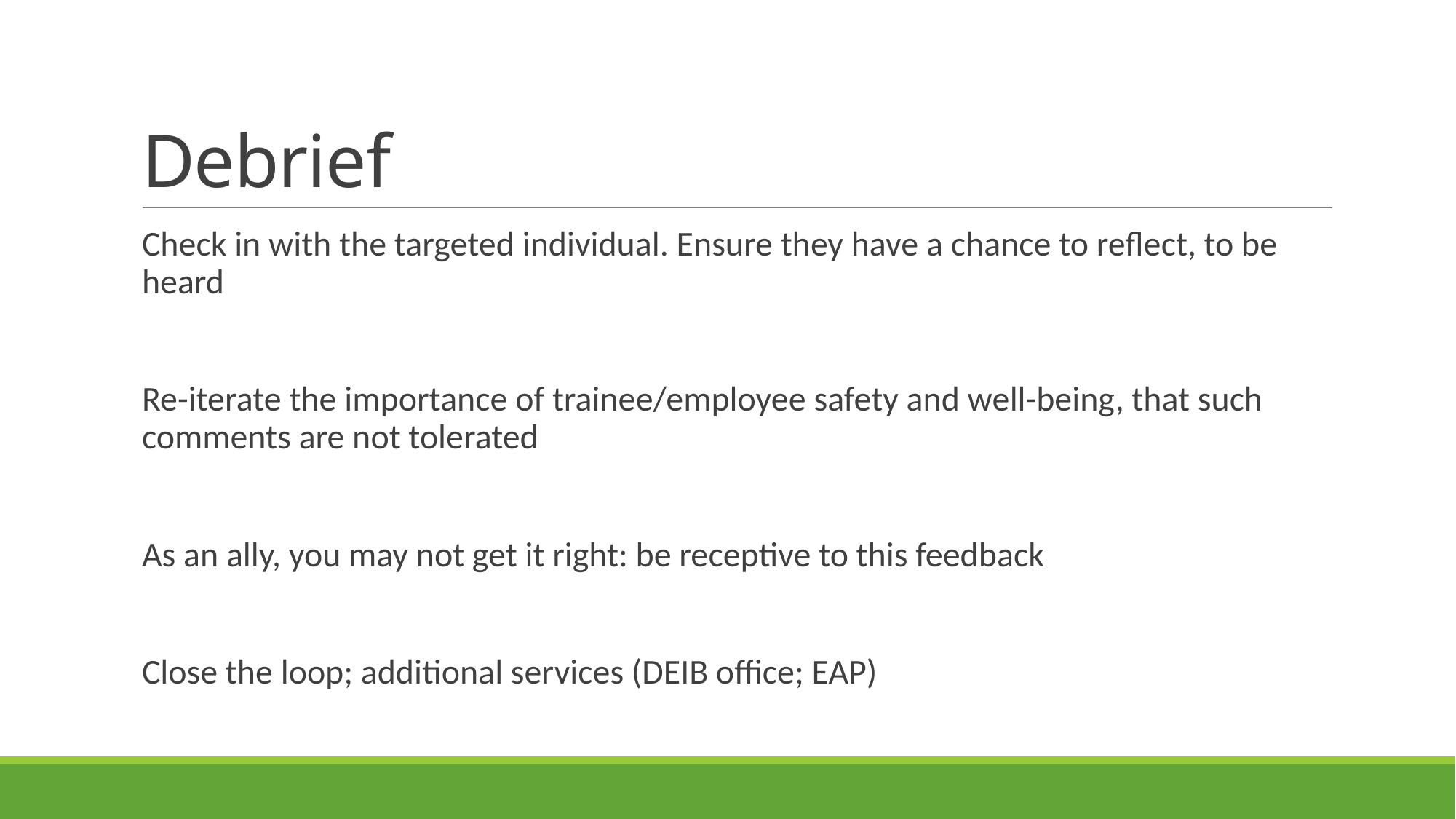

# Debrief
Check in with the targeted individual. Ensure they have a chance to reflect, to be heard
Re-iterate the importance of trainee/employee safety and well-being, that such comments are not tolerated
As an ally, you may not get it right: be receptive to this feedback
Close the loop; additional services (DEIB office; EAP)

## Slide 25
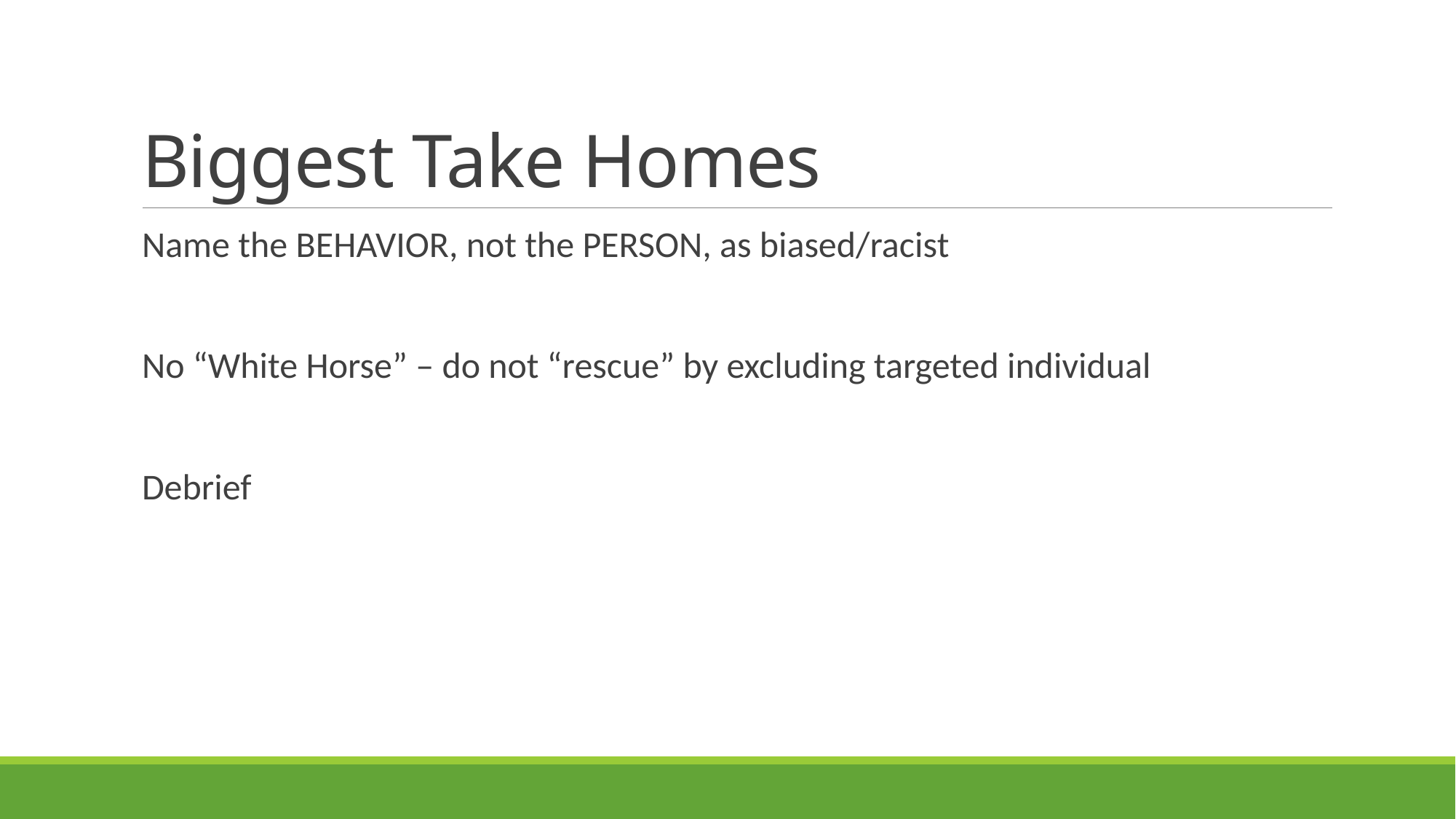

# Biggest Take Homes
Name the BEHAVIOR, not the PERSON, as biased/racist
No “White Horse” – do not “rescue” by excluding targeted individual
Debrief

## Slide 26
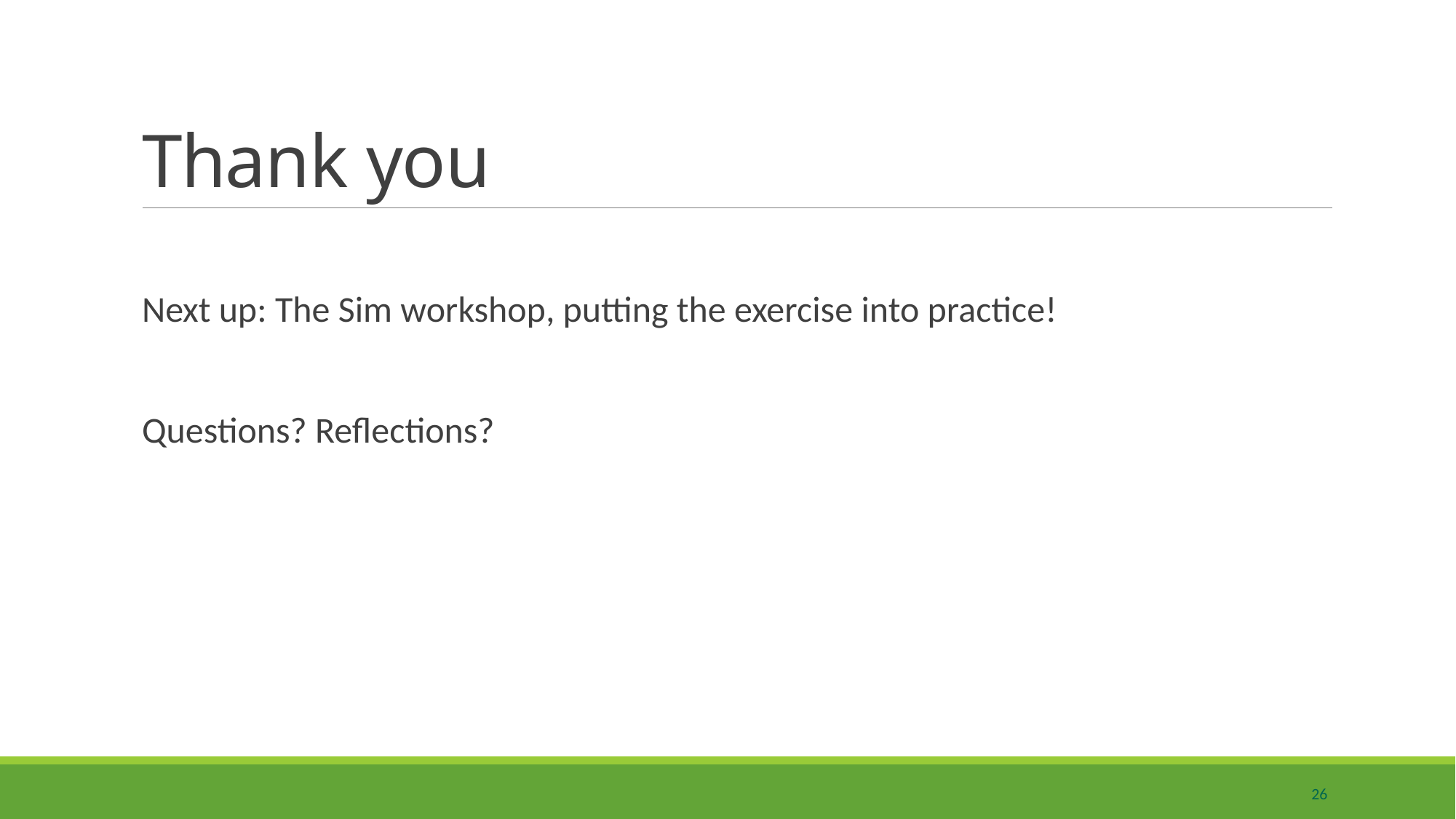

# Thank you
Next up: The Sim workshop, putting the exercise into practice!
Questions? Reflections?
26

## Slide 27
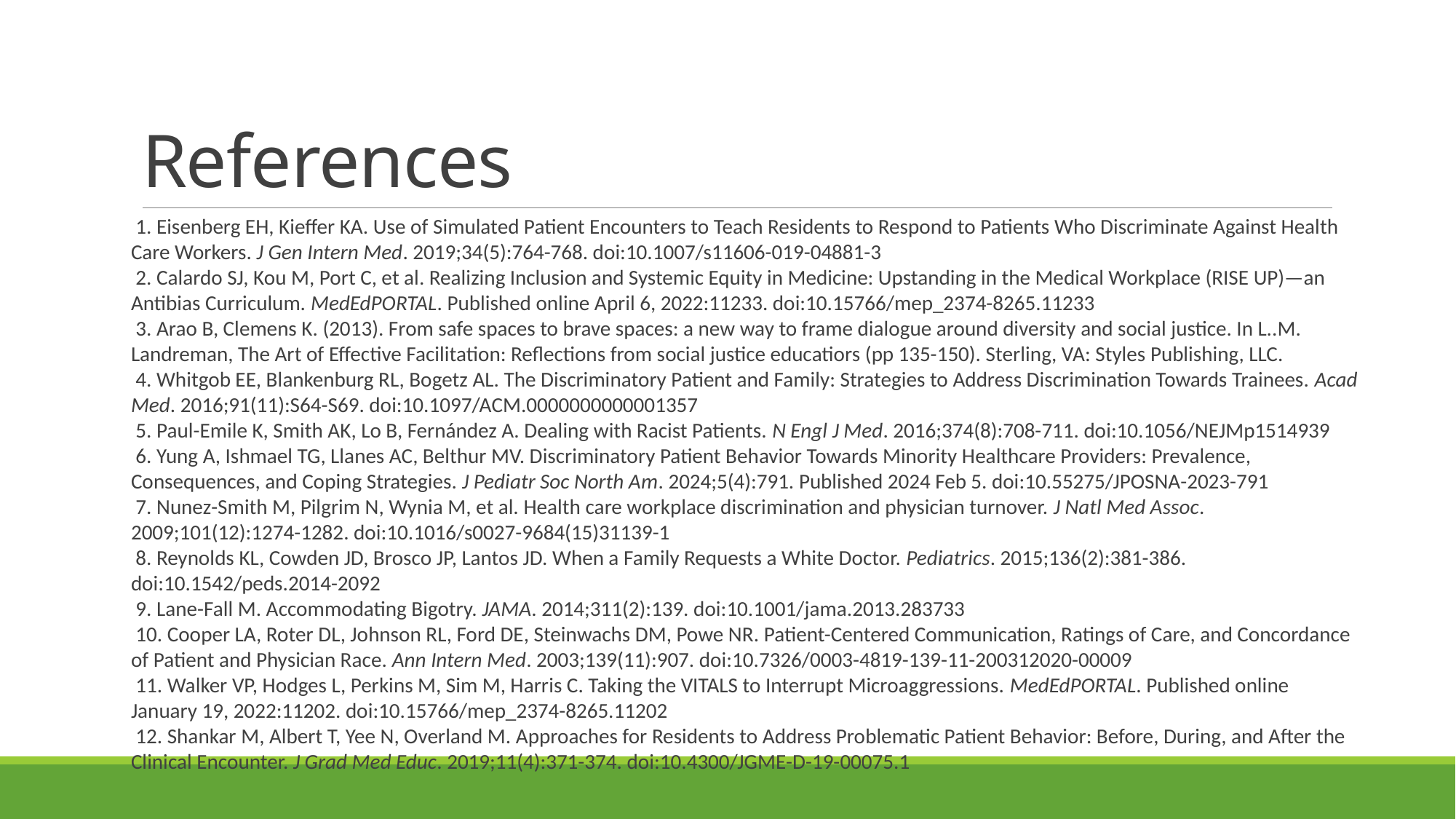

# References
1. Eisenberg EH, Kieffer KA. Use of Simulated Patient Encounters to Teach Residents to Respond to Patients Who Discriminate Against Health Care Workers. J Gen Intern Med. 2019;34(5):764-768. doi:10.1007/s11606-019-04881-3
2. Calardo SJ, Kou M, Port C, et al. Realizing Inclusion and Systemic Equity in Medicine: Upstanding in the Medical Workplace (RISE UP)—an Antibias Curriculum. MedEdPORTAL. Published online April 6, 2022:11233. doi:10.15766/mep_2374-8265.11233
3. Arao B, Clemens K. (2013). From safe spaces to brave spaces: a new way to frame dialogue around diversity and social justice. In L..M. Landreman, The Art of Effective Facilitation: Reflections from social justice educatiors (pp 135-150). Sterling, VA: Styles Publishing, LLC.
4. Whitgob EE, Blankenburg RL, Bogetz AL. The Discriminatory Patient and Family: Strategies to Address Discrimination Towards Trainees. Acad Med. 2016;91(11):S64-S69. doi:10.1097/ACM.0000000000001357
5. Paul-Emile K, Smith AK, Lo B, Fernández A. Dealing with Racist Patients. N Engl J Med. 2016;374(8):708-711. doi:10.1056/NEJMp1514939
6. Yung A, Ishmael TG, Llanes AC, Belthur MV. Discriminatory Patient Behavior Towards Minority Healthcare Providers: Prevalence, Consequences, and Coping Strategies. J Pediatr Soc North Am. 2024;5(4):791. Published 2024 Feb 5. doi:10.55275/JPOSNA-2023-791
7. Nunez-Smith M, Pilgrim N, Wynia M, et al. Health care workplace discrimination and physician turnover. J Natl Med Assoc. 2009;101(12):1274-1282. doi:10.1016/s0027-9684(15)31139-1
8. Reynolds KL, Cowden JD, Brosco JP, Lantos JD. When a Family Requests a White Doctor. Pediatrics. 2015;136(2):381-386. doi:10.1542/peds.2014-2092
9. Lane-Fall M. Accommodating Bigotry. JAMA. 2014;311(2):139. doi:10.1001/jama.2013.283733
10. Cooper LA, Roter DL, Johnson RL, Ford DE, Steinwachs DM, Powe NR. Patient-Centered Communication, Ratings of Care, and Concordance of Patient and Physician Race. Ann Intern Med. 2003;139(11):907. doi:10.7326/0003-4819-139-11-200312020-00009
11. Walker VP, Hodges L, Perkins M, Sim M, Harris C. Taking the VITALS to Interrupt Microaggressions. MedEdPORTAL. Published online January 19, 2022:11202. doi:10.15766/mep_2374-8265.11202
12. Shankar M, Albert T, Yee N, Overland M. Approaches for Residents to Address Problematic Patient Behavior: Before, During, and After the Clinical Encounter. J Grad Med Educ. 2019;11(4):371-374. doi:10.4300/JGME-D-19-00075.1
