# Supplementary material for: Responding to Families Who Express Biases: An Adaptable Standardized Participant Communication Simulation to Train Upstander Pediatric Providers
Source: MedEdPORTAL. 2026 Mar 27;22:11588. doi: 10.15766/mep_2374-8265.11588 (PMC13021565; doi:10.15766/mep_2374-8265.11588)
Supplement: Supplementary file 1 — Scripted Language Tool.docxCase 1 - Inpatient.docxCase 2 - Inpatient.docxCase 3 - Inpatient_SP1.docxCase 3 - Inpatient_SP2.docxCase 3 - Simulation.docxFacilitator Guide.docxSP Educator Training Notes.docxAnti-bias Intro Presentation.pptxPre- and Postsurveys.docx [file mep_2374-8265.11588-s001.zip › J. Pre- and Postsurveys.docx]

**Presurvey.** Distribute to participants during the prebrief.

Please indicate your level of agreement with each of the following statements.

Note: “Discriminatory behavior” refers to any biased behavior, including macro- and microaggressions, that focuses on recipient’s race, gender, ethnicity, religion, culture, etc.

1. Discriminatory behavior is **prevalent** in the clinical environment

| Strongly agree | Agree | Neutral | Disagree | Strongly Disagree |
| --- | --- | --- | --- | --- |

1. I have personally **witnessed** discriminatory behavior by a parent/family-member of a pediatric patient and had to navigate the experience in front of the pediatric patient:

| Never | A few times | Monthly | Weekly | Daily |
| --- | --- | --- | --- | --- |

1. I have the **tools and language** to respond to discriminatory behavior in the workplace/clinical setting.

| Strongly agree | Agree | Neutral | Disagree | Strongly Disagree |
| --- | --- | --- | --- | --- |

1. I feel **confident navigating** parental/family discriminatory behavior while modeling appropriate behavior in front of pediatric patients.

| Strongly agree | Agree | Neutral | Disagree | Strongly Disagree |
| --- | --- | --- | --- | --- |

1. Prior to today, have you received training on managing discriminatory behavior from families?

| Yes | No |
| --- | --- |

**Postsurvey.** Distribute to participants at the conclusion of the debrief.

Please indicate your level of agreement with each of the following statements.

Note: “Discriminatory behavior” refers to any biased behavior, including macro- and microaggressions, that focuses on recipient’s race, gender, ethnicity, religion, culture, etc.

1. The **quality** of the training workshop was high.

| Strongly agree | Agree | Neutral | Disagree | Strongly Disagree |
| --- | --- | --- | --- | --- |

1. The training was **relevant** to my professional development and work.

| Strongly agree | Agree | Neutral | Disagree | Strongly Disagree |
| --- | --- | --- | --- | --- |

1. I learned a **new skill** during this training.

| Strongly agree | Agree | Neutral | Disagree | Strongly Disagree |
| --- | --- | --- | --- | --- |

1. I have the **tools and language** to respond to discriminatory behavior in the workplace/clinical setting.

| Strongly agree | Agree | Neutral | Disagree | Strongly Disagree |
| --- | --- | --- | --- | --- |

1. I feel **confident** navigating parental/family discriminatory behavior while modeling appropriate behavior in front of pediatric patients.

| Strongly agree | Agree | Neutral | Disagree | Strongly Disagree |
| --- | --- | --- | --- | --- |

1. What were the strengths of this activity? (Open response)
2. How can this activity be improved? (Open response)
